# Predation of boreal owl nests by pine martens in the boreal forest does not vary as predicted by the alternative prey hypothesis

**DOI:** 10.1007/s00442-022-05149-0

**Published:** 2022-03-19

**Authors:** Geir A. Sonerud

**Affiliations:** Faculty of Environmental Sciences and Natural Resource Management, Norwegian University of Life Sciences, P. O. Box 5003, NO-1432 Ås, Norway

**Keywords:** *Aegolius funereus*^.^, Alternative prey hypothesis, Cavity-nesting, Long-term spatial memory, *Martes martes*, Microtine rodents, Nest predation

## Abstract

The alternative prey hypothesis (APH) states that temporally synchronous population fluctuations of microtine rodents and other small herbivores are caused by generalist predators that show functional and numerical responses to the abundance of microtines. This would lead to an increased predation of alternative prey in the low phase of the microtine population fluctuations. One candidate for such a predator is the tree-climbing pine marten (*Martes martes*), which includes bird eggs in its diet, among them eggs of the cavity-nesting boreal owl (*Aegolius funereus*). I used long-term data to test whether pine marten predation of boreal owl eggs in nest boxes varied as predicted by the APH. The probability of predation of owl nests situated < 45 km from a site where microtines were trapped in spring during four decades increased with microtine trapping index, which is opposite to the prediction from the APH. As the data set was limited to one nest per box, I extended it spatially and temporally using the clutch size of each boreal owl nest as a proxy for the actual microtine abundance at the site. The probability of nest predation increased with clutch size. However, the effects of microtine index and owl clutch size became non-significant when I controlled for habitat, and in particular cavity age, which had an overriding effect. The increase in predation probability with cavity age suggests that the long-term spatial memory of pine marten is an important factor in the pattern of its nest predation in tree cavities.

**Supplementary Information:**

The online version contains supplementary material available at 10.1007/s00442-022-05149-0.

## Introduction

According to the alternative prey hypothesis (APH), the temporally synchronous 3–4 year periodic population fluctuations of microtine rodents and other small herbivores, in particular species of grouse, in Fennoscandia are caused by generalist predators showing functional and numerical responses to the abundance of microtine rodents, their main prey (Hagen 1952; Angelstam et al. 1984, 1985). This would lead to an increased predation of grouse and other prey, the predators’ alternative prey, in the low phase of the microtine rodent population fluctuations (Hagen 1952; Angelstam et al. 1984, 1985). The APH is equivalent to explanations for predator–prey relationship and prey population dynamics in resource pulse-driven systems in general, based on functional response in predators and effects on alternative prey (Schmidt and Ostfeld 2008).

The two generalist predator species that have been regarded as most important in the APH are the red fox (*Vulpes vulpes*) and the pine marten (*Martes martes*). In predator removal experiments, including both red foxes and pine martens, the effect of each species could not be disentangled (Marcström et al. 1988, 1989; see also Lindström et al. 1987; Kurki et al. 1997). Population changes following an irruption of the sarcoptic mange (*Sarcoptes scabei*) in Sweden and Norway in the 1970s revealed that the effect of the red fox on alternative prey was according to the APH (Lindström et al. 1994). It also revealed that the red fox limited the pine marten population (Lindström et al. 1995; Smedshaug et al. 1999). The pine marten population was negatively affected by the red fox in the same way as those of grouse and mountain hare (*Lepus timidus*), and the positive correlations between the hunting bags of pine marten and grouse and mountain hare suggested that the pine marten was a less important predator on grouse than the red fox (Smedshaug et al. 1999). Thus, whether pine marten predation on alternative prey depends on the size of microtine rodent populations, as predicted by the APH, has not been established.

Whereas the effect of red fox on the population dynamics of willow grouse (*Lagopus lagopus*) was recently confirmed to be as predicted by the APH (Breisjøberget et al. 2018), there are no studies clearly demonstrating this effect of pine marten on grouse. Jahren et al. (2017) found that pine marten predation on black grouse (*Tetrao tetrix*) nests declined with increasing microtine rodent density when the pine marten density was low and medium, but increased with increasing vole density when pine marten density was high. Pine marten predation on capercaillie (*Tetrao urogallus*) nests was not as predicted by the APH, but increased with pine marten density. Jahren et al. (2017), therefore, suggested that woodland grouse nests are alternative prey for the red fox, but not for the pine marten, and that the marten seems to be a more specialized nest predator than the fox.

The temporally synchronous fluctuations in grouse and microtine rodents, although consistent with the prediction from the APH, may also be due to fluctuations in the quality of their common food (Selås 2006, 2019; Selås et al. 2011). Thus, the APH should be tested for alternative prey other than the herbivorous grouse and mountain hare (Selås 2006). Because the tree-climbing pine marten preys on bird eggs in general, including those located in tree cavities, studying its effect on nests of other birds than grouse presents an opportunity to test the generality of the APH independently of fluctuations in the quality of the small herbivores´ food.

Boreal owls (*Aegolius funereus*) occur over large parts of the Holarctic boreal forest (Cramp 1985). In the western Palearctic, they nest mostly in cavities excavated by the black woodpecker (*Dryocopus martius*) (e.g. Cramp 1985), but they readily accept nest boxes (e.g. Sonerud 1985a). They are exposed to a significant risk of nest predation from the pine marten (Sonerud 1985a, b, 1989, 1993; Johnsson 1993; Zarybnicka et al. 2015a). Pine martens are medium-sized (c. 1 kg) mustelids with relatively large home ranges (on average 7 km^2^ at 60°N in Sweden and Norway) and a generalist diet (Brainerd 1997; Helldin 1999). They visit tree cavities year-round and use them for roosting, denning and food storing (Sonerud 1985b; Brainerd et al. 1995), and take any prey that happen to be there, including eggs and nestlings. The positions of cavities are probably learned (Sonerud 1985a, 1989, 1993), and pine martens spend most time on the ground and prey mainly on small mammals, in Fennoscandia microtine rodents (Pulliainen and Ollimäki 1996; Helldin 2000). Pine martens prefer older forest and avoid open habitats such as clear-cuts (Brainerd and Rolstad 2002, cf. Sonerud 1985b).

At northern latitudes, boreal owls show a strong numerical response to microtine rodents (Hörnfeldt et al. 1990; Korpimäki and Hakkarainen 1991; Zarybnicka et al. 2015b). In the frame of the APH, this makes the relationship between pine marten, microtine rodents and predation on boreal owl nests less straightforward than the relationship between pine marten, microtine rodents and predation on nests of alternative prey species that have no numerical response to microtine rodents, such as birds that nest every year (Pöysä et al. 2016). In Norway, pine marten predation on boreal owl nests seemed to be independent of the microtine rodent abundance, but the relationship was difficult to untangle, because the boreal owls rarely nested in years with low microtine abundance (Sonerud 1985a). In contrast, in the Czech Republic, Zarybnicka et al. (2015a) found that pine marten predation on boreal owl nests was inversely related to the abundance of *Apodemus* mice. In Finland, Pöysä et al. (2016) found that predation by pine marten on nests of the cavity-nesting common goldeneye (*Bucephala clangula*) did not vary with the microtine rodent abundance as predicted by the APH, and suggested that this may be due to individual martens learning the nest box locations. Because the results from previous studies in Fennoscandia on pine marten predation of nests in tree cavities have not supported the APH (Sonerud 1985a; Pöysä et al. 2016), and because this may be due to an important trait in the predatory behavior of the pine marten, viz. its spatial memory of tree cavities (Sonerud 1985a, 1989, 1993), I included this as a control factor in the analysis. In addition, I included habitat as a control factor, due to the pine marten’s affinity to habitats with forest cover (Brainerd and Rolstad 2002).

Here, I extend the studies of Zarybnicka et al. (2015a) and Pöysä et al. (2016) by demonstrating that pine marten spatial memory of nest boxes overrides any effect of microtine abundance on the probability of nest predation. First, according to the APH, the probability of pine marten predation of a boreal owl nest should be lower when microtine rodents are abundant than when they are scarce. Second, the probability of pine marten predation of boreal owl nests in nest boxes has been found to increase with time since the box was installed, a pattern attributed to pine martens memorizing the spatial position of nest boxes they have found and revisiting them in later breeding seasons (Sonerud 1985a, 1989, 1993). Third, because pine martens prefer older forest and avoid open habitats such as clear-cuts (Brainerd and Rolstad 2002), one would expect the probability of pine marten predation of a boreal owl nest to decline with distance from the forest into clear-cuts. Thus, I predicted that the probability of pine marten predation of a boreal owl nest in a nest box would decline with increasing microtine rodent abundance, increase with time elapsed since the nest box had been installed, and decline with distance from forest into clear-cuts. I tested these three predictions using long-term data on pine marten predation on boreal owl eggs located in nest boxes in Norway.

## Materials and methods

### Study area

**Fig. 1:**
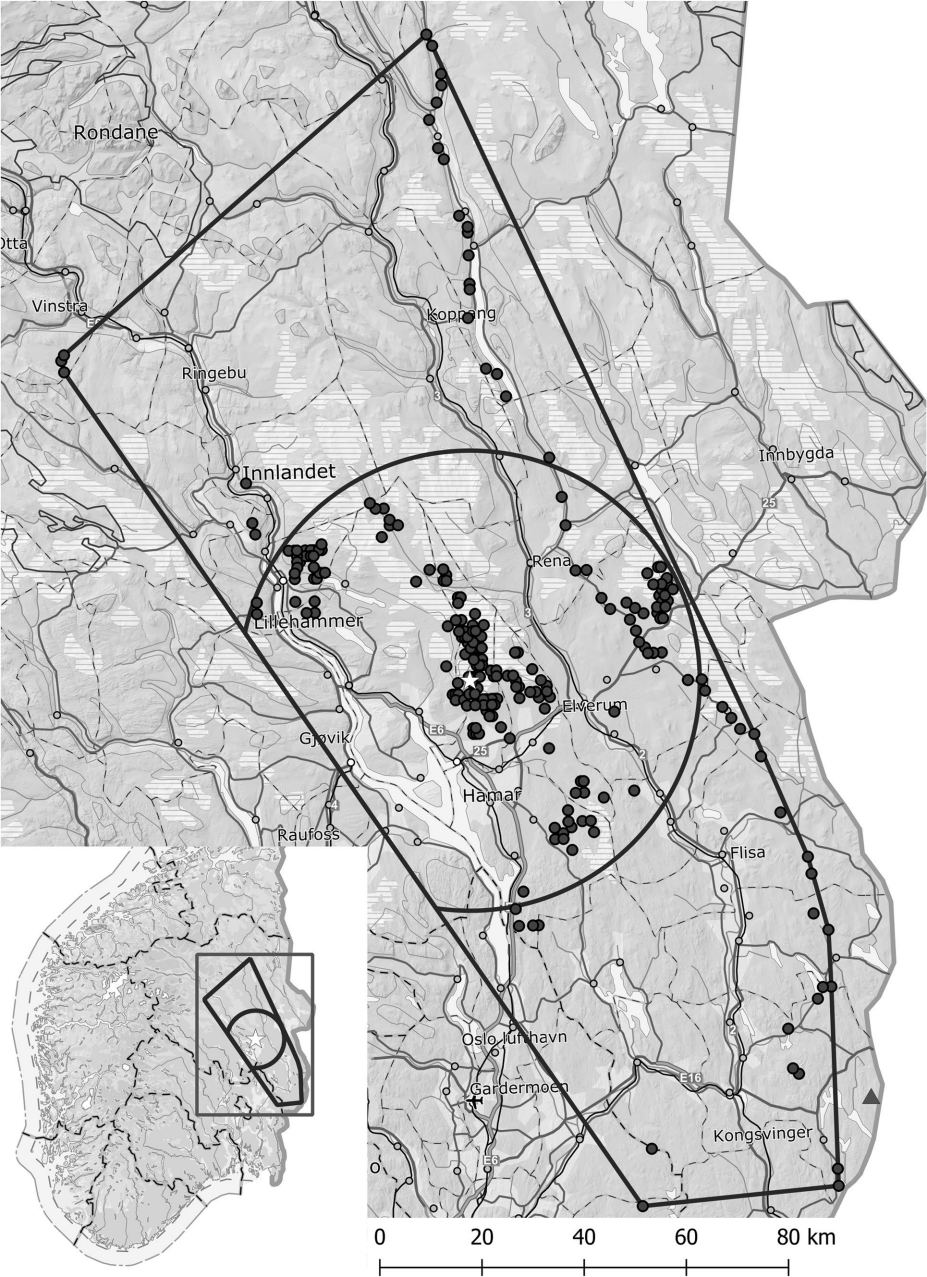
Map of southeast Norway showing the extent of the study area as a minimum convex polygon including the boxes used by boreal owl (filled circles), the site where microtine rodents were trapped (open star), and a circle with radius 45 km around the microtine rodent trapping site. A microtine rodent trapping site (Wegge and Rolstad 2018) outside the study area is shown by a filled triangle. In the cases where two or more nest boxes were closer to each other than 500 m only one is shown

The study was conducted during 1970–2018 in the boreal zone within 60°00′–62°04′ N and 9°40′–12°23′ E in Hedmark and Oppland counties (from 2020 combined to Innlandet County) in southeast Norway (Fig. 1). The study area is covered by coniferous forest managed by modern forestry techniques, i.e. harvesting by unselective clear-cutting, regeneration by planting, and thinning by selective cutting. It includes the study areas of Sonerud (1985a, b) and Steen et al. (1996), and additional areas further west. The convex polygon circumscribing the nest boxes spans an area ca. 16,000 km^2^ (Fig. 1). The elevation of the nest boxes ranged 170–890 m, with median = 455 m and mean ± se = 475 ± 8 m (*n* = 340).

### Study species

Boreal owls are small (male body mass ca. 100 g) and nocturnal and subsist mainly on small mammals (Cramp 1985). Due to the strong numerical response of boreal owls to microtine rodents at northern latitudes (Hörnfeldt et al. 1990; Korpimäki and Hakkarainen 1991; Zarybnicka et al. 2015b), very few territories in my study area support nesting each year, and at most only 1–2 nestings in each 3–4 year microtine population cycle (Sonerud 1985a). Adult males are usually locally resident, whereas adult females may disperse widely between successive nesting attempts in response to the spatially asynchronous 3–4 year population fluctuations of microtine rodents (e.g. Löfgren et al. 1986; Korpimäki et al. 1987; Sonerud et al. 1988). Nest-site selection seems to depend mostly on the male (Hakkarainen and Korpimäki 1998; Sonerud 2021), who provides all prey for the family as long as the female incubates and broods the nestlings, and most or all prey thereafter until the young become independent (Eldegard and Sonerud 2009, 2010, 2012). The eggs are laid with ca. 2 day intervals, incubation starts with the first egg, and each egg is incubated for ca. 29 days, although somewhat shorter with increasing number in the laying sequence; ca. 27 days for the last egg in an average clutch of five eggs (Korpimäki 1981). The first-fledged nestling in each nest fledges at an age of 29–36 days, on average 33 days (Eldegard and Sonerud 2012).

### Nest boxes

Nest boxes were installed most years. Most boxes were installed ca. 5 m above ground. In comparison, cavities excavated by the black woodpecker in Sweden and Norway were on average ca. 7 m above ground (Johnsson et al. 1993; Rolstad et al. 2000). When installed, all boxes were lined with a 5–10 cm deep layer of fine wood shavings covering the bottom. Boxes were not systematically cleaned after each nesting.

Most boxes were installed in single trees in clear-cut areas or other open habitats, or in trees in edges between old forest and clear-cuts or other habitats, and fewer in the interior of old forest stands. This reflected the habitat preferences of black woodpeckers selecting a tree in which to excavate a nesting cavity (see Rolstad et al. 2000) and made the boxes attractive for boreal owls as well (cf. Sonerud 1985b). Of the 340 boxes used for nesting by boreal owls in this study, 146 were situated in a clear-cut or another open habitat, 128 in the edge between old forest and clear-cuts or other open habitats, and 66 within an old forest stand.

### Nests

Each box was usually visited several times between March and July each year to record the onset and outcome of boreal owl nesting attempts (date of egg laying, clutch size, and whether the nest was predated). I defined a box as being selected when at least one boreal owl egg had been laid there. An already recorded boreal owl nest was scored as predated when either all eggs had been removed, or broken eggs or eggshells were found (see also Sonerud 1985a, b).

Because almost all predation occurred before hatching (see Results), and because it was sometimes hard to separate between predation of nestlings and abandonment of the brood due to, for instance, insufficient prey abundance, I restricted scoring the probability of predation to the time period from the first egg was laid until all eggs had hatched. This also avoided any effect of brood size on the probability of predation through noise from nestlings during prey deliveries.

Because I knew all potential nest sites before each season, I was able to also record nests that were predated prior to the first check, minimizing the problem of underestimating nest predation by failure to include nests already predated (see Sonerud 1985a, b). Still, if a nest had been predated prior to the first nest box check for the season, and all eggs had been removed, the nesting attempt may have been overlooked rather than scored as predated. Thus, the probability of nest predation is inherently underestimated. Because some boreal owl nests were abandoned without being predated, I avoid using the term successful nests as a contrast to predated nests, and rather use the term nests that escaped predation.

I was able to score whether the nest was predated or not for a total of 540 boreal owl nests exposed to nest predation for which there were data on habitat and cavity age (see below). Of these, 133 were included in a previous study on the effect of cavity age on nest predation (Sonerud 1985a).

The probability of predation of a boreal owl nest in a nest box has been found to depend on whether the previous boreal owl nest in the same box had been predated or not (Sonerud 1985a). Therefore, to minimize the dependence between data points in the analysis, I used only one nest per nest box, either the only one, or the first one. Of the 340 boxes in this study, 221 had only one boreal owl nest, and 119 had 2–9 nests (Electronic Supplementary Material 1 (ESM 1), Table S1). Of the 217 boxes situated < 45 km from the microtine trapping site (Fig. 1) during the years when microtine rodents were trapped (1977–78 and 1981–2018; see below), 145 had only one boreal owl nest, and 72 had 2–6 nests (ESM 1, Table S1).

In the data set from the boxes situated < 45 km from the microtine rodent trapping site during the trapping years 1977–78 and 1981–2018, the first nest in a box from 1977 and onwards was included in the analysis, independent of whether there had been any boreal owl nest in the same box prior to 1977. Thus, for the years 1977–78 and 1981–85 the number of nests included in the analysis based on nests < 45 km from the microtine trapping site was larger than the number of nests included in the analysis based on the whole study area, which also included the years 1970–76 (see ESM 1, Table S2).

### Microtine rodent abundance

In 1977–1978 and 1981–2018, I trapped microtine rodents at the same site each spring as soon as the snow cover had disappeared, which varied from early May to early June. The trapping site was situated in the boreal forest at an elevation of 550–600 m at 60°56´N, 11°08´E (Fig. 1, see also Sonerud (1986)). In each trapping session, I put out c. 300 wooden snap traps (brand Rapp) baited with cocoa fat (brand Delfia) and checked them each morning for 4 days. The traps were set c. 5 m apart in seven separate lines > 160 m apart within an area of c. 40 ha. The lines were kept the same through all 40 trapping years while the forest cover changed. Most traps were in a clear-cut at the start and in middle-aged forest at the end, although the others were in old forest at the start and in plantations or young forest at the end (see Sonerud (1986, 1988a) for a description of the trapping area in the first years of the trapping).

Number of trap nights each spring ranged 1000–1184, with median = 1095 and mean ± se = 1096 ± 6 (*n* = 40). I calculated a microtine rodent trapping index as number of animals of all recorded microtine species (bank vole (*Myodes glareolus*), field vole (*Microtus agrestis*), tundra vole (*Microtus oeconomus*) and wood lemming (*Myopus schisticolor*)) pooled trapped per 100 trap nights. This index ranged over two orders of magnitude (0.10–8.04). I also calculated a corresponding index separately for bank vole, *Microtus* voles, and wood lemming. As an index of the change in microtine abundance since the previous year, I subtracted the trapping index of the previous year from the trapping index of the current year.

The microtine rodent abundance fluctuated quite regularly during the first two decades of my trapping, with an interval of 3–4 years between peaks (ESM 2, Fig. S1). However, the peaks became gradually lower, and an expected peak in 2001 did not appear (ESM 2, Fig. S1). From 2005 and onwards, the pattern with distinctive peaks reappeared, and the last three peaks were even higher than the peaks during the first two decades (ESM 2, Fig. S1). Thus, the microtine rodent population fluctuations were overall as required for testing the APH.

Population fluctuations of microtine rodents tend to be spatially synchronized over large areas, but the extent of this synchronization is poorly documented. A study performed along a gradient of almost 300 km along the east side of my study area (see Fig. 1) during 1990–1994 found that local populations of bank vole up to 30–40 km apart exhibited statistically significant synchrony in growth patterns (Steen et al. 1996). It has later turned out that this estimate was obtained during a period with generally low amplitudes and low spatial synchrony of bank vole populations in Hedmark County (Selås et al. 2021). Prior to 1990 and after 2003 bank vole populations at two sites in Hedmark County located 120 km apart (my trapping site and that of Wegge and Rolstad (2018); see Fig. 1) fluctuated in synchrony (Selås et al. 2021). Because almost all the cases where boreal owls used nest boxes situated 35–45 km from my microtine trapping site occurred either prior to 1990 or after 2003, I used 45 km as a conservative limit for microtine abundance at my trapping site being representative (see Fig. 1).

### Boreal owl clutch size as proxy of microtine rodent abundance

To increase the sample size both in time and space, and to include boreal owl nests recorded in years when I did not trap microtines (1970–76 and 1979–80), as well as boreal owl nests situated > 45 km from the trapping site (see Fig. 1), I used boreal owl clutch size as a proxy for microtine abundance. For the sample of all boreal owl nests recorded < 45 km from the microtine rodent trapping site during 1977–78 and 1981–2018 for which the clutch size was known (*n* = 223), i.e. not only one nest per box, clutch size increased significantly with microtine rodent trapping index (ESM 1, Table S3, ESM 2, Fig. S2). A corresponding numerical response of boreal owls has been found elsewhere in Fennoscandia (e.g. Hörnfeldt et al. 1990; Korpimäki and Hakkarainen 1991; Zarybnicka et al. 2015b). Therefore, I used the clutch size of each boreal owl female recorded nesting as a proxy of the microtine abundance in the home range of her mate during the period (egg laying and incubation) for which the occurrence of predation was scored. In the cases where predation had occurred before the first check, or before the clutch was recorded as complete, I assigned the clutch size of the nearest recorded neighbor nest in the same year. For three nests, this was not possible because all known nests in the actual year were predated (one nest in 1986 and two nests in 2008). When limiting the data set to one nest per box, the distance to the nearest neighbor in the remaining 78 cases, where I used the clutch size of the nearest recorded neighbor nest in the same year, ranged 0.3–42 km, with median = 3.0 km and mean ± se = 6.5 ± 1.1 km.

For the nests where I was able to score whether predation occurred or not, and was able to score habitat and cavity age (see below), the assigned clutch size ranged 2–10, with median = 5 and mean ± se = 5.3 ± 0.06 (*n* = 337).

### Predator identification

The predator was identified from marks in the broken eggs, hairs in the box entrance, scats on the roof of the box, or tracks in the snow. Among the 145 cases of recorded predation, 100 were attributed to pine marten, and 45 to an unidentified predator. In the latter cases, however, the pine marten could not be excluded as predator (see also Sonerud 1985a, b). I therefore assume that all cases of nest predation were due to pine marten.

When restricting the sample to one nest per nest box, there were 76 cases of recorded predation, of which 53 were attributed to pine marten, and 23 to an unidentified predator. The probability that a case of predation was attributed to an unidentified predator was not significantly affected by the microtine rodent abundance (ESM 1, Table S4a, ESM 2, Fig. S3a), by the change in microtine abundance from the previous spring to the current spring (ESM 1, Table S4b, ESM 2, Fig. S3b), or by the proxy for microtine rodent abundance, the boreal owl clutch size (ESM 1, Table S4c, ESM 2, Fig. S3c). In fact, the weak trend indicated a lower probability of identifying the predator as pine marten with lower microtine abundance, with smaller increase in microtine abundance since the previous spring, and with lower clutch size as a proxy for microtine abundance. This suggests that, if anything, the real pine marten predation in the microtine low years may have been even lower than estimated. Thus, any error in assuming that all cases of predation were due to pine marten would be conservative with respect to testing the APH.

### Pine marten abundance

An epizootic of sarcoptic mange among red foxes spread from the first cases in central Norway in 1975–1976 to the whole country during the next 10 years, resulting in a severe decline of the red fox population (Smedshaug et al. 1999; cf. Lindström et al. 1994). This led to an increase in the hunting bags of its prey species capercaillie, black grouse and mountain hare, and also of the pine marten (Smedshaug et al. 1999), the latter probably due to relaxed competition and predation from the red fox (Lindström 1989; Storch et al. 1990; Lindström et al. 1995). In my study area, the peak effect of the red fox reduction on the harvest of the prey species seemed to be reached around 1990 (Smedshaug et al. 1999, ESM 2, Fig. S4). Thereafter, the red fox population recovered (Selås 1998; cf. Breisjøberget et al. 2018), and hunting bags of the prey species, including the pine marten, decreased (Selås 1998, see below).

The population density of the pine marten may affect the probability of pine marten nest predation (cf. Jahren et al. 2017). Therefore, I extracted data on pine marten hunting bags from Statistics Norway (2020). Smedshaug et al. (1999) and Statistics Norway (2020) describe in more detail how these data are compiled from the hunters’ reports. The pine marten hunting bag from one season consists of all animals harvested from 1 November in year *N-1* to 15 March in year *N*. Among the pine martens harvested, almost all are trapped and very few shot. On the national level, data are available from 1972 and onwards (49 years), and the annual hunting bag ranged 1600–11,300, with median 4420 (ESM 2, Fig. S4). On the regional level, data are available for a shorter period, for Hedmark and Oppland counties from 1992 and onwards (29 years), and the annual hunting bag here ranged 560–1690, with median 790 (ESM 2, Fig. S4). Although there was a fairly good association between the trend in annual marten harvest number on the regional level (Hedmark and Oppland counties) and the national level (*R*^*2*^ = 0.40, *n* = 29), there was a low association between the year-to-year fluctuations (detrended series) at the regional and national level (*R*^*2*^ = 0.04, *n* = 28). Because the regional series was too short for my purpose, and because the national series was poorly associated with the regional one from year to year, I refrained from using the pine marten hunting bag as a variable in the analyses, except for descriptive purpose.

### Cavity age

I scored cavity age for a nest box as the number of nesting seasons elapsed since the box had been installed in the actual tree, assigning the value 1 for the first nesting season the box was available, 2 for the second season, and so on. For the first (or only) boreal owl nest in a box, cavity age ranged from 1 to 27 for the 340 nesting attempts for which I knew the cavity age and habitat and was able to score whether predation occurred or not. For one of these nests (0.3%) cavity age exceeded 15. For this nest, cavity age was truncated to 15 in the analyses. Then, cavity age ranged 1–15, with median = 2 and mean ± se = 2.8 ± 0.14 (*n* = 340).

### Habitat

Pine martens in the boreal forest of Norway and Sweden prefer habitats with continuous tree canopy (hereafter forest) and avoid habitats without, particularly clear-cut areas (Brainerd and Rolstad 2002). Therefore, as a linear measure of habitat relevant for the probability of predation of a nest in a box I simply used the shortest distance from the box to the nearest edge between forest and an open habitat, the latter being either a clear-cut area or a bog. Nest boxes situated at the edge between forest and open habitat were assigned a value of zero, whereas boxes situated within a forest stand were assigned a negative value and boxes situated in a tree in a clear-cut or a bog were assigned a positive value. Among the 340 nest boxes used by boreal owls in this study for which I knew the cavity age (see above) and habitat, and was able to score whether predation occurred or not, the distance to the forest edge ranged from −270 to 150 m. This distance exceeded 100 m in five cases (1.5%), three cases for boxes in open habitats (positive values) and two for boxes in habitats with forest canopy (negative values). For these cases, distance to forest edge was truncated to 100 and −100 m, respectively. Then, distance to forest edge ranged −100 to 100 m, with median = 0 m and mean ± se = 3 ± 1 m (*n* = 340).

### Statistical analyses

Data preparation and explorative analyses were conducted in JMP® Pro version 15.0.0 (SAS 2019), and the final analyses were performed using general linear mixed models (GLMM) in package `lme4` in R version 4.0.3 (R Core Team 2020). For all models, the `bobyqa` optimizer was used to avoid mild non-convergence (source code provided in ESM 3). The response variable was whether a nest was scored as predated or not (binomial distribution). In models with nests situated < 45 km from the microtine rodent trapping site in a year when I trapped microtine rodents, fixed explanatory variables were microtine rodent trapping index, cavity age, and distance from the nest box to the nearest edge between forest and open habitats. In models with nests from the whole study area and all study years, microtine rodent trapping index was substituted with boreal owl clutch size as a proxy.

Correspondingly, when testing the effect of uncertainty in predator identification, the response variable was whether a predated nest was scored as taken by pine marten or by an unidentified predator. In the model with nests situated < 45 km from the microtine rodent trapping site, the fixed explanatory variable was microtine rodent abundance, whereas in the model with all nests, the fixed variable was boreal owl clutch size as a proxy for microtine rodent abundance.

When estimating the effect of microtine rodent abundance on boreal owl clutch size (Poisson distribution), all recorded boreal owl nest situated < 45 km from the microtine trapping in the years that I trapped microtine rodents were included, i.e. also repeated nests in the same nest box.

Year was conservatively added as a random effect in all models to determine whether it explained any deviation. This was not the case (SD = 0) in the models where I tested the effect of the change in microtine index from the previous year to the current year (187 nests, 33 years), where all variation was allocated to the fixed effect of microtines.

In each analysis, models with all combinations of the explanatory variables and their interactions were created with the dredge function in R (package ‘MuMin’). Candidate models were ranked using the Akaike information criterion corrected for small sample size (AICc), following recommendations by Burnham et al. (2011) and Richards et al. (2011). I considered models with ΔAICc < 2.0 to be well supported and thus competing with the model with lowest AICc value. Among competing models, the one with the lowest number of effects was considered the most parsimonious. I also report AICc weight for all models, and use evidence ratio (ER) when comparing some of the models, i.e. the ratio between the corresponding AICc weights (see Burnham et al. (2011), Richards et al. (2011) and Cade (2015) for definitions). I followed the advice by Cade (2015) and refrained from model averaging as well as the use of relative weight of a variable (i. e. the sum of AICc weights for all models in a model set in which the variable appeared) for evaluating the relative importance of explanatory variables.

Fixed explanatory variables were standardized. In all models, all correlations between fixed variables were < 0.30. For the most parsimonious and the highest-ranked models, I provide parameter estimates. All estimates are given with ± 1 se. For further analysis of a significant interaction between three fixed variables, package ‘ggeffects’ in R was used. Figures were made in JMP® Pro version 15.0.0 and in R using base functions and package `ggplot2`.

## Results

### Overall nest predation

For the first or only nest in a box, the overall probability of predation of a boreal owl nest prior to hatching of all eggs was 0.22 (*n* = 340). In addition to these 76 cases of predation, another six nests were predated at the nestling stage. Thus, among the 82 identified cases of predation, 93% occurred during incubation. In another 14 cases where the nest escaped predation until hatching was complete, I was unable to score whether or not predation had occurred during the nestling stage. Overall probability of predation was then 0.25 (*n* = 326).

**Fig. 2:**
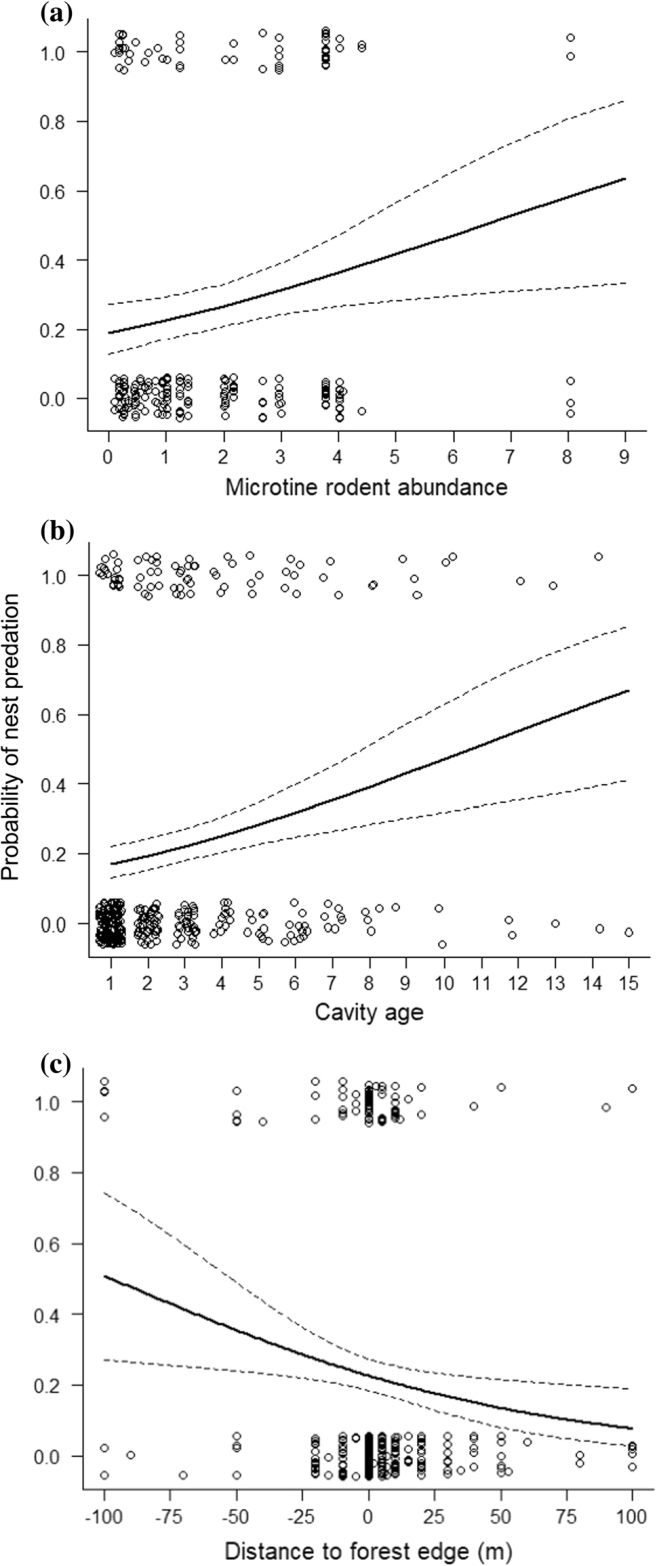
The probability of predation of a boreal owl nest as function of the three main fixed variables, with data from one nest per nest box. **a** Microtine rodent spring trapping index (*n* = 217, slope = 0.224 ± 0.090, *z* = 2.490, *P* = 0.013). **b** Cavity age (*n* = 340, slope = 0.164 ± 0.045, *z* = 3.624, *P* = 0.0003). **c** Distance to forest edge (*n* = 340, slope = −0.0127 ± 0.00511, *z* = −2.476, *P* = 0.013)

The probability of nest predation increased with increasing microtine rodent trapping index (Fig. 2a), increased with increasing cavity age (Fig. 2b), and declined with increasing distance from the forest interior across the forest edge and further into open habitats (Fig. 2c). The increase in nest predation with increasing microtine abundance is opposite to the prediction from the APH. The probability of nest predation tended to decline with increasing pine marten hunting bag in Norway (ESM 2, Fig. S5).

### Effect of microtine rodent abundance

Among the models including microtine rodent trapping index, cavity age, and distance to forest edge, the most parsimonious model included only cavity age (ESM 1, Table S5). In this model, the effect of cavity age was highly significant, and the probability of nest predation increased with increasing cavity age (Table 1a). A model also including distance to forest edge had higher AIC weight (ESM 1, Table S5; evidence ratio (ER) = 1.46). In this model, the effect of cavity age was highly significant, while the effect of distance to forest edge was not significant (Table 1b). The full model had the highest AIC weight (ESM 1, Table S5), but only slightly higher than the model with only cavity age and distance to forest edge (ER = 1.02). In the full model, the effect of cavity age was highly significant, while the only other significant effect was the three-way interaction between microtine index, cavity age, and distance to forest edge (Table 1c). For nests in habitat with forest cover, the probability of predation increased with cavity age when microtine abundance was medium or high, but not when it was low (ESM 2, Fig. S6). For nests at or near the forest edge, the probability of predation increased with cavity age for all levels of microtine abundance (ESM 2, Fig. S6). For nests in open habitat far from the edge to forest, the probability of predation increased with cavity age when microtine abundance was low or medium, but not when it was high (ESM 2, Fig. S6). Apart from the full model, the highest-ranked model with microtine trapping index included had the fourth highest AIC weight (ESM 1, Table S5). In this model, the effect of cavity age was highly significant and the effect of distance to forest edge marginally non-significant, while the effect of microtine abundance was not significant (Table 1d).

**Table 1:** Parameter estimates in a subset of models for the probability of predation of a boreal owl nest in relation to microtine rodent abundance in spring the same year (*n* = 217, corrected for the random effect of 35 years)

Explanatory variable	Estimate ± SE	*z*	*P*
*a*			
Intercept	−1.311 ± 0.238	−5.509	< 0.0001
Cavity age	0.726 ± 0.170	4.260	< 0.0001
*b*			
Intercept	−1.303 ± 0.235	−5.549	< 0.0001
Cavity age	0.716 ± 0.171	4.188	< 0.0001
Distance to edge	−0.302 ± 0.186	−1.624	0.10
*c*			
Intercept	−1.308 ± 0.236	−5.548	< 0.0001
Microtines	0.164 ± 0.220	0.743	0.46
Cavity age	0.614 ± 0.181	3.389	0.0007
Distance to edge	−0.229 ± 0.207	−1.105	0.27
Cavity age * distance to edge	−0.088 ± 0.198	−0.445	0.66
Microtines * cavity age	0.044 ± 0.206	0.214	0.83
Microtines * distance to edge	0.087 ± 0.236	0.369	0.71
Microtines * cavity age * distance to edge	−0.919 ± 0.376	−2.442	0.015
*d*			
Intercept	−1.240 ± 0.212	−5.863	< 0.0001
Microtines	0.186 ± 0.199	0.938	0.35
Cavity age	0.686 ± 0.173	3.976	< 0.0001
Distance to edge	−0.313 ± 0.189	−1.684	0.092

*a* The most parsimonious model (AICc = 229.9). *b* The second most parsimonious model (AICc = 229.2). *c* The full model (AICc = 229.1). *d* The simplest model with microtine rodent trapping index included (AICc = 230.5). All models included in this analysis are described and compared in Table S5

Generalized linear mixed-effect models with log link function, binomial distribution, and Adaptive Gause-Hermite quadrature approximation to the likelihood. Continuous explanatory variables are standardized. The boreal owl nests were situated < 45 km from the microtine rodent trapping site

Because the microtine index was a pooled sample of the microtine species trapped, with a different ratio between the species in different years, and because the probability of being trapped may differ between the species, I performed an alternative analysis using the same fixed variable as above, but substituting the microtine index with a separate index for bank vole, *Microtus* voles (field vole and tundra vole pooled), and wood lemming. In all three cases, the most parsimonious model included only cavity age (ESM 1, Tables S6-S8). In the highest-ranked model with the trapping index for the actual microtine species included, the effect of cavity age was highly significant, while the effect of the actual microtine species abundance was not significant (ESM 1, Table S9). The simple separate effects of the abundance of bank vole, *Microtus* voles, and wood lemming, respectively, on the probability of nest predation are shown in ESM 2, Fig. S7.

### Effects of change in microtine abundance since previous year

Nest predation by pine marten may be affected more by the change in microtine abundance from the previous year than by the current abundance. According to the APH, a decline in microtine abundance would increase the probability of nest predation, while an increase in microtine abundance would decrease the probability of nest predation. I therefore repeated the tests above, substituting the current microtine trapping index with the change in trapping index from the previous year. This inevitable reduced the sample size, but did not change the main results.

Among the models including the change in microtine rodent trapping index from the previous year, cavity age, and distance to forest edge, the most parsimonious model, which also was the model with highest AIC weight, included only cavity age (ESM 1, Table S10). In this model, the effect of cavity age was highly significant, and the probability of predation increased with increasing cavity age (Table 2a). A model also including distance to forest edge had the second highest AIC weight (ESM 1, Table S10; evidence ratio (ER) = 2.21). In this model, the effect of cavity age was highly significant, while the effect of distance to forest edge was not significant (Table 2b). The highest-ranked model with change in microtine trapping index included had the third highest weight (ESM 1, Table S10). In this model, the effect of cavity age was highly significant, while the effect of change in microtine abundance and the effect of distance to forest edge were not significant (Table 2c). The simple separate effect of the change in the abundance of microtines from the previous year on the probability of nest predation is shown in ESM 2, Fig. S8a.

**Table 2:** Parameter estimates in a subset of models for the probability of predation of a boreal owl nest in relation to the year-to-year change in the microtine rodent abundance in spring (*n* = 187, corrected for the random effect of 33 years)

Explanatory variable	Estimate ± SE	*z*	*P*
*a*			
Intercept	−1.438 ± 0.192	−7.490	< 0.0001
Cavity age	0.514 ± 0.165	3.111	0.0019
*b*			
Intercept	−1.442 ± 0.193	−7.489	< 0.0001
Cavity age	0.508 ± 0.166	3.071	0.0021
Distance to edge	−0.135 ± 0.193	−0.699	0.48
*c*			
Intercept	−1.438 ± 0.192	−7.490	< 0.0001
Microtines change	0.003 ± 0.181	0.015	0.99
Cavity age	0.514 ± 0.165	3.107	0.0019
*d*			
Intercept	−1.442 ± 0.193	−7.489	< 0.0001
Microtines change	0.016 ± 0.184	0.085	0.93
Cavity age	0.508 ± 0.166	3.064	0.0022
Distance to edge	−0.136 ± 0.193	−0.703	0.48

*a* The most parsimonious model (AICc = 185.4). *b* The second most parsimonious model (AICc = 187.0). *c* The simplest model with change in microtine rodent trapping index included (AICc = 187.5). *d* The simplest model with change in microtine rodent trapping index, cavity age, and distance to forest edge included (AICc = 189.1). All models included in this analysis are described and compared in Table S10

Generalized linear mixed-effect models with log link function, binomial distribution, and Adaptive Gause-Hermite quadrature approximation to the likelihood. Continuous explanatory variables are standardized. The boreal owl nests were situated < 45 km from the microtine rodent trapping site

Also among the models that included the change in trapping index from the previous year separately for bank vole, *Microtus voles*, and wood lemming, the most parsimonious model, which also was the model with highest AIC weight, included only cavity age (ESM 1, Tables S11-S13). In the highest-ranked model with the change in trapping index for the actual microtine species included, the effect of cavity age was highly significant, while the effect of the change in actual microtine species abundance was not significant (ESM 1, Table S14). The simple separate effects of the change in the abundance of bank vole, *Microtus* voles, and wood lemming, respectively, from the previous year on the probability of nest predation are shown in ESM 2, Figs. S8b–d.

### Boreal owl clutch size as a proxy for microtine rodent abundance

To test the APH also for boreal owl nests situated > 45 km from the microtine rodent trapping site, and from years that I did not trap microtines (1970–76 and 1979–80), I used boreal owl clutch size as a proxy for microtine rodent abundance. First, to see how representative this proxy was, I substituted microtine abundance with boreal owl clutch size for nests situated < 45 km from the trapping in the years when I trapped microtine rodents. The most parsimonious model included only cavity age (ESM 1, Table S15). The highest-ranked model with clutch size included had the fourth highest weight (ESM 1, Table S15). In this model, the effect of cavity age was highly significant, while the effect of clutch size and the effect of distance to forest edge were not significant (ESM 1, Table S16). Thus, the effect of clutch size was similar to the effect of microtines (cf. Table 1d), with a positive effect of both (Fig. 2a and ESM 2, Fig. S8a).

Based on the finding above that boreal owl clutch size substituted well for microtine rodent trapping index in explaining the probability of nest predation, I used it as a proxy for microtine rodent abundance for all boreal owl nests in my study. Then, the most parsimonious model, which also was the model with highest weight, included cavity age and distance to forest edge, while clutch size was included in the model with the third highest weight (ESM 1, Table S17). In the latter model, the effect of clutch size was not significant, while the effect of distance to forest edge was significant, and the effect of cavity age highly significant (ESM 1, Table S18). The simple separate effect of boreal owl clutch size as a proxy for microtine rodent abundance on the probability of nest predation is shown in ESM 2, Fig. S8b.

### Minimizing the effect of cavity age

Because the analyses above showed that cavity age measured on an annual scale had a major effect on the probability of nest predation, I eliminated its effect by only including nests found in a box the first season the box was available. This inevitably reduced the sample size. Whether microtine abundance was taken as the spring trapping index, the change in trapping index from the previous to the current spring, or the boreal owl clutch size as a proxy, the null model performed better than any model based on one of these variables and the distance to forest edge (ESM 1, Tables S19–S21). In the models where the effect of distance from forest edge was controlled for, the effect of microtine abundance was not significant (ESM 1, Tables S22–S24).

## Discussion

I found no support for the APH in my data on predation of boreal owl nests by the pine marten. First, the effect of the recorded microtine rodent abundance on the probability of nest predation was opposite to that predicted from the APH. It became weaker when I controlled for distance to forest edge, and in particular cavity age, but remained positive. As an alternative analysis, I substituted the trapping index of microtine rodents pooled with the trapping index of bank vole, *Microtus* voles, and wood lemming separately. However, this did not change the results qualitatively; the effect of the microtines was negligible, and for bank vole and *Microtus* voles opposite to that predicted from the APH.

Second, a decline in microtine abundance should, according to the APH, increase the probability of nest predation by pine marten, while an increase in microtine abundance would decrease the probability of nest predation. However, the effect of a change in microtine abundance on the probability of nest predation was negligible.

Third, using the clutch size of each nesting boreal owl pair (or in the case it was unknown due to predation, the clutch size of its nearest recorded neighbor) as an indirect measure of the microtine abundance at the time of the nesting, I was able to utilize an extended dataset on predation of boreal owl nests covering a larger area and more years. However, neither in the extended dataset was pine marten predation of boreal owl nests affected by microtine abundance as predicted by the APH.

The microtine trapping index, as well as the proxy for microtine abundance, namely the clutch size of each recorded boreal owl nest, was, if anything, positively related to the probability of predation. This indicates a higher probability of predation with higher microtine abundance in spring, which is opposite to the prediction by the APH. Pöysä et al. (2016) found a corresponding trend for pine marten predation of common goldeneye nests, and attributed it to a higher abundance of juvenile pine martens due to a higher survival during winters with high microtine abundance.

Increasing predation of boreal owl nests with increasing clutch size may theoretically be due to longer exposure period, because the time elapsed from laying of the first egg until hatching of the last egg increases with clutch size. The interval between laying of successive eggs in the boreal owl is 2 days, and each egg is incubated for ca. 29 days, somewhat shorter with higher number in the laying sequence (Korpimäki 1981). Thus, the time from laying of the first egg until all eggs have hatched would be ca. 31 days for a clutch of two eggs, ca. 34 days for a clutch of five eggs and ca. 37 days for a clutch of eight eggs (see Methods). Compared to this increase, the mean nestling period of 33 days for the first-fledged offspring (Eldegard and Sonerud 2012) is c. five times longer. Among predated nests, > 90% were taken before hatching was complete, compared with < 10% during the similarly long nestling period. Thus, the increase in probability of nest predation did not scale linearly to the number of days exposed, whereas a linear scaling would be expected if the pine marten encountered nest boxes by random. This suggests that a substantial proportion of predation occurred either because most boxes happened to be installed where the pine marten innately traveled often (site effect sensu Martin et al. 2000), or that the pine marten already had learned the position of most boxes and revisited them regularly. The latter is supported by the fact that the probability of nest predation increased with cavity age (cf. Sonerud 1985a).

In contrast to my finding from Norway for the boreal owl and that of Pöysä et al. (2016) from Finland for the common goldeneye, where pine marten nest predation was independent of microtine rodent abundance, Zarybnicka et al. (2015a) found that pine marten predation of boreal owl nests in the Czech Republic was inversely related to the abundance of *Apodemus* mice. The difference may be explained if the way that boreal owl and pine marten depend on their main prey is different when main prey are microtine rodents than when main prey are *Apodemus* mice. In the temperate forests of Central Europe, pine marten and boreal owl show functional response to *Apodemus* mice in spring, but not to microtines (Jedrzejewski et al. 1993; Zarybnicka et al. 2013). For the boreal owl, a higher abundance of prey alternative to *Apodemus* in the Czech Republic than of prey alternative to microtines in Fennoscandia may allow boreal owls to nest at a lower density of the main prey in the temperate forests in the Czech Republic than in the boreal forest in Fennoscandia (Zarybnicka et al. 2015b). Thus, lack of data from years with really low microtine abundance in the boreal forest may explain the difference between boreal and temperate forest in the pattern of pine marten predation on boreal owl nests. However, Pöysä et al. (2016) did not find elevated predation of common goldeneye nests in the boreal forest in years with low microtine abundance.

The boreal owl nests on which I based my indirect estimate of the microtine rodent abundance by applying the clutch size as proxy were in most cases (77%) the actual boreal owl nest, and in the remaining cases (23%) the nearest boreal owl neighbor nest because the actual nest had been predated before the clutch size could be recorded. In these cases, the median neighbor distance was 3 km, 75% were shorter than 6 km, 90% shorter than 17 km, and none longer than 42 km. I therefore regard my indirect estimate of the microtine abundance to be spatially precise. Also, I regard it to be temporally precise, reflecting the abundance of microtines when the boreal owls were nesting, unless the microtine population underwent a crash after the boreal owls had completed their clutch, but before all eggs had hatched.

There was an overriding effect of cavity age on the probability of predation of boreal owl nests. This would confirm the pattern of increasing probability of predation with cavity age previously found for boreal owls, explained as pine martens memorizing the spatial position of nest boxes they have found and revisiting them in later breeding seasons (Sonerud 1985a). Boreal owls minimize this nest predation by preferring new cavities (Sonerud 1985a, 2021). Note that because I used only the first boreal owl nest in each box, the increasing probability of nest predation with increasing cavity age was not due to the pine marten returning to boxes where it had taken a boreal owl nest previously. In fact, this result suggests a pattern where a pine marten finds more boxes as the years pass by, and once a box has been found it is revisited later. Similarly, the fact that a boreal owl nest in a box where the previous nest had escaped predation was more likely to be predated if the previous nest was 2 years ago than if it was 1 year ago, was interpreted as the pine marten finding boxes also when they were empty and including them in later foraging trips (Sonerud 1985a). Hence, the simple pattern that a box where a nest has been predated is profitable to revisit and an empty box is unprofitable to revisit is not applicable to pine marten nest predation in boxes.

When I only included nests in boxes the first season they were available I minimized the effect of cavity age on the probability of nest predation. Still, there was only a negligible effect of microtine abundance. This suggests that the probability that a pine marten encounters a new box, and thus the extent of a pine marten´s foraging trips, was not affected by the microtine abundance. The nest boxes were installed between early autumn and early spring. Therefore, a small effect of spatial memory cannot be excluded, even for new boxes, as the pine marten may have found some of them during fall and winter, and revisited them in the first breeding season.

Although second to cavity age, the habitat in which the cavity was situated, expressed as distance to the edge between open habitats, mostly clear-cuts, and habitats with forest canopy, mostly mature forest, had an effect of the probability of nest predation. Independent of cavity age, the probability of depredation tended to decline from the interior of forest stands 100 m from forest edge to the same distance from forest edge into open habitats. Similarly, for the black woodpecker the probability of pine marten nest predation was higher in mature forest than in clear-cuts (Rolstad et al. 2000). Studies based on radio telemetry in boreal forests in Norway and Sweden found that pine martens prefer habitats with forest canopy and tall trees, and avoid clear-cuts and other open areas, although they are able to utilize a wide range of succession stages of the forest (Brainerd and Rolstad 2002). The most important predator on the pine marten is the red fox (Lindström et al. 1995), which in the boreal forest, although being a habitat generalist, prefers clear-cuts and other open areas (Storch et al. 1990).

The significant effect of the interaction between cavity age, distance to forest edge, and microtine abundance may suggest that the pine marten foraged to a higher extent in the preferred habitat with forest cover when microtine abundance was high than when it was low, and was forced to forage more in the non-preferred open habitat when microtine abundance was low.

In Fennoscandia, the red fox limits the pine marten population (Lindström et al. 1995; Smedshaug et al. 1999). The red fox shows a numerical response to microtine rodents, resulting in a one-year lag from the peak in the microtine rodent population to the peak in the red fox population (Lindström 1982). Thus, the effects of red fox on pine marten, both directly through predation and indirectly through any effect of “landscape of fear” (e.g. Lindström et al. 1995; Lyly et al. 2015), would probably be largest in the year after a microtine rodent population peak, although the additional red foxes in such years are young individuals with limited hunting experience (cf. Sonerud 1988b). The pine marten population in the year following a peak in the microtine population may therefore be lower than otherwise expected from any numerical response to microtine rodents alone. This may be an additional explanation for the fact that pine marten predation of boreal owl nests did not increase with declining microtine rodent abundance.

In conclusion, the APH hypothesis was refuted by my data on pine marten predation of boreal owl nests. Overall, the best predictor of the probability of predation of a boreal owl nest was cavity age, suggesting that pine martens revisited nest boxes they had once found, no matter the microtine abundance. This adds to the awareness of the importance of learning and spatial memory in the behavior of nest predators in particular (Sonerud 1985a, c; Sonerud and Fjeld 1987; Pelech et al. 2010; Pöysä et al. 2016), and predators in general (Mitchell and Lima 2002).

## Supplementary Information

Below is the link to the electronic supplementary material.

Supplementary file1 (PDF 169 KB)

Supplementary file2 (PDF 619 KB)

Supplementary file3 (TXT 20 KB)

## Data Availability

GAS conceived, designed, and executed this study and wrote the manuscript. No other person is entitled to authorship.

## Availability of data and material

The datasets used and analyzed during the current study are available from the author on reasonable request.

## Code availability

The code is provided in supplementary material.
