# Supplementary material for: Predation of boreal owl nests by pine martens in the boreal forest does not vary as predicted by the alternative prey hypothesis
Source: Oecologia. 2022 Mar 19;198(4):995–1009. doi: 10.1007/s00442-022-05149-0 (PMC9056444; doi:10.1007/s00442-022-05149-0)
Supplement: Supplementary file 1 — Supplementary file1 (PDF 169 KB) [file 442_2022_5149_MOESM1_ESM.pdf]

Electronic Supplementary Material (ESM 1)

Sonerud GA (2022) Predation of boreal owl nests by pine martens in the boreal forest does not vary as predicted by the alternative prey hypothesis

Tables S1 - S24

Oecologia

Geir A. Sonerud

Faculty of Environmental Sciences and Natural Resource Management

Norwegian University of Life Sciences

P. O. Box 5003, NO-1432 Ås

Norway

E-mail: geir.sonerud@nmbu.no

Table S1 Number of boreal owl nests distributed on number of nest boxes used.

	All nests (1970 - 2018)		Nests situated < 45 km from trapping site in trapping years (1977-78 and 1981-2018)	
Nests per box	Boxes	Nests	Boxes	Nests
1	221	221	145	145
2	77	154	46	92
3	20	60	14	42
4	11	44	9	36
5	8	40	2	10
6	2	12	1	6
7	0	0	0	0
8	0	0	0	0
9	1	9	0	0
Total	340	540	217	331

Table S2 Annual number of boreal owl nests (total and predated) in the whole study area and in the area < 45 km from the microtine rodent trapping site, when only one nest per nest box is included. CavAge is the mean number of nesting seasons since the nest box was installed. DistEdge is the mean distance from the actual nest box to the edge between old forest and clear-cuts and other open habitats, with negative values for boxes in forest and positive for boxes in open habitats. MicIndex is microtine rodent trapping index in spring. For nest boxes with more than one nest during the study, only the first nest is included, separately for the whole study area during the whole study period, and for the area <45 km from the microtine rodent trapping site during the years when microtine rodents were trapped. Nest situated < 45 km from the microtine trapping site in 1970-76 and 1979-80 are not shown because no microtine trapping took place these years.

	Whole study area				< 45 km from microtine trapping site				
Year	Total	Pred	CavAge	DistEdge	Total	Pred	CavAge	DistEdge	MicIndex
1970	1	0	1	0	-	-	-	-	-
1971	3	0	1.0	10	-	-	-	-	-
1972	3	1	1.7	-17	-	-	-	-	-
1973	11	3	2.1	-2	-	-	-	-	-
1974	34	9	2.3	-10	-	-	-	-	-
1975	0	0	-	-	-	-	-	-	-
1976	7	1	2.7	-21					
1977	12	6	3.3	-4	27	16	4.4	-5	3.78
1978	5	3	4.4	19	6	3	4.5	16	0.25
1979	0	0	-	-	-	-	-	-	-
1980	5	2	6.0	2	-	-	-	-	-
1981	7	3	5.3	-6	11	6	6.0	-13	2.96
1982	0	0	-	-	0	0	-	-	0.09
1983	0	0	-	-	0	0	-	-	0.55
1984	2	0	1.0	8	3	1	5.0	7	0.85
1985	16	4	2.3	5	16	5	3.1	5	1.24
1986	1	1	2	0	1	1	2	0	0.37
1987	8	0	2.4	17	8	0	2.4	17	1.39
1988	11	2	2.9	18	9	2	2.8	21	2.68
1989	4	1	2.8	-3	4	1	2.8	-3	0.27
1990	5	1	2.8	7	5	1	2.8	7	0.35
1991	32	5	1.7	8	13	1	2.3	11	1.02
1992	22	6	2.0	15	9	2	2.1	16	2.18
1993	15	5	1.7	0	5	1	1.2	9	0.29
1994	7	1	3.1	4	2	0	5.5	0	0.95
1995	13	2	2.2	4	7	1	2.4	4	0.93
1996	8	1	2.6	-6	4	1	3.3	-3	0.10
1997	11	2	2.6	-21	5	1	4.6	0	0.62
1998	15	3	1.9	9	10	1	2.3	10	0.47
1999	5	0	2.0	21	3	0	1.7	4	0.45

	Whole study area				< 45 km from microtine trapping site				
Year	Total	Pred	CavAge	DistEdge	Total	Pred	CavAge	DistEdge	MicIndex
2000	5	1	2.4	4	5	1	2.4	4	0.19
2001	6	2	1.8	8	6	2	1.8	8	0.18
2002	3	0	2.7	-7	3	0	2.7	-7	0.26
2003	4	0	3.5	1	4	0	3.5	1	0.55
2004	5	1	4.6	0	4	1	5.5	-2	0.70
2005	11	1	2.4	5	9	1	2.7	6	2.04
2006	5	0	2.6	6	3	0	3.0	10	0.82
2007	4	0	5.3	8	2	0	3.5	23	0.27
2008	2	2	6.5	3	2	2	6.5	3	0.19
2009	1	1	12	12	1	1	12	12	0.19
2010	4	2	8.5	-5	3	2	9.7	-7	4.41
2011	14	2	5.9	-1	14	2	5.9	-1	4.02
2012	0	0	-	-	0	0	-	-	0.19
2013	2	0	4.0	20	2	0	4.0	20	3.01
2014	5	2	3.8	30	5	2	3.8	30	8.04
2015	0	0	-	-	0	0	-	-	0.29
2016	0	0	-	-	0	0	-	-	0.46
2017	1	0	4.0	40	1	0	4	40	4.07
2018	5	0	5.0	30	5	0	5.0	30	2.00
Total	340	76	2.8	3	217	58	3.7	6	

Table S3 Parameter estimates from the model for clutch size of a boreal owl nest (*n* = 223) as a fixed effect of the microtine rodent trapping index in spring, corrected for the random effect of year (*n* = 34).

Explanatory variable	Estimate ± SE	z	*P*
Intercept	1.594 ± 0.042	38.389	< 0.0001
Microtine rodent abundance	0.041 ± 0.017	2.512	0.012

Generalized linear mixed-effect model with log link function, Poisson distribution, and Laplace approximation to the likelihood.

Table S4 Parameter estimates for the probability that the predator of a predated boreal owl nest was scored as pine marten rather than as an unidentified predator as an effect of a fixed variable, corrected for the random effect of year. **a** Fixed effect is the microtine rodent trapping index in spring (60 nests, 25 years). **b** Fixed effect is the change in microtine rodent abundance from the previous spring to the current spring (51 nests, 23 years). **c** Fixed effect is the size of the actual boreal owl clutch predated or the size of the clutch of the nearest neighbor, taken as a proxy for the microtine rodent abundance (73 nests, 28 years).

Explanatory variable	Estimate ± SE	z	*P*
**a**			
Intercept	1.086 ± 0.441	2.464	0.014
Microtine rodent abundance	0.019 ± 0.381	0.051	0.96
**b**			
Intercept	1.121 ± 0.490	2.288	0.022
Change in microtine rodent abundance	0.134 ± 0.457	0.293	0.77
**c**			
Intercept	1.045 ± 0.345	3.029	0.0025
Boreal owl clutch size	0.187 ± 0.285	0.654	0.51

Generalized linear mixed-effect model with log link function, binomial distribution, and Adaptive Gause-Hermite quadrature approximation to the likelihood. Explanatory variables are standardized.

Table S5 Model selection based on Akaike´s information criterion (AIC) to determine which variables influenced the probability of predation of a boreal owl nest situated < 45 km from the microtine rodent trapping site in a year when microtine rodents were trapped (*n* = 217), with year as random effect (*n* = 35). MicRod (range 0.10 – 8.04) denotes the microtine rodent spring trapping index (see text for details). Cav (range 1 – 15) denotes the time since the actual nest box was installed (see text for details). Dist (range -100 – 100) denotes the shortest distance from the nest box to the nearest edge between forest and an open habitat (see text for details). Models are sorted by their degree of support from the data, as indicated by the AICc weight (w). Models with ΔAIC ≤ 2.0 from the best model are shown in bold.

Variables	LogLikelihood	df	AICc	ΔAIC	AIC w
**Cav + Dist + MicRod + Cav*Dist + Cav*MicRod + Dist*MicRod + Cav*Dist*MicRod**	**-105.14**	**9**	**229.1**	**0.00**	**0.207**
**Cav + Dist**	**-110.50**	**4**	**229.2**	**0.05**	**0.202**
**Cav**	**-111.92**	**3**	**229.9**	**0.81**	**0.138**
**Cav + Dist + MicRod**	**-110.09**	**5**	**230.5**	**1.32**	**0.107**
**Cav + Dist + Cav*Dist**	**-110.19**	**5**	**230.7**	**1.52**	**0.097**
Cav + MicRod	-111.62	4	231.4	2.28	0.066
Cav + Dist + MicRod + Cav*Dist	-109.79	6	232.0	2.85	0.050
Cav + Dist + MicRod + Dist*MicRod	-110.03	6	232.5	3.32	0.039
Cav + Dist + MicRod + Cav*MicRod	-110.08	6	232.6	3.42	0.037
Cav + MicRod + Cav*MicRod	-111.60	5	233.5	4.34	0.024
Cav + Dist + MicRod + Cav*Dist + Dist*MicRod	-109.77	7	234.1	4.94	0.017
Cav + Dist + MicRod + Cav*Dist + Cav*MicRod	-109.79	7	234.1	4.98	0.017

Table S6 Model selection based on Akaike´s information criterion for small sample size (AICc) to determine which variables influenced the probability of predation of a boreal owl nest (*n* = 217) situated < 45 km from the microtine rodent trapping site in a year when microtine rodents were trapped (1977-78 and 1981-2018), with year as random effect (*n* = 35). BankVole (range 0.00 – 3.10) denotes the bank vole spring trapping index (see text for details). Cav (range 1 – 15) denotes the time since the actual nest box was installed, with 1 denoting the first nesting season the box was available (see text). Dist (range -100 – 100) denotes the shortest distance from the nest box to the nearest edge between forest and an open habitat, with zero for boxes situated at the edge between forest and open habitat, a negative value for boxes situated within a forest stand, and a positive value for boxes situated in a tree in a clear-cut or a bog (see text for details). Models are sorted by their degree of support from the data, as indicated by the AICc weight (w). Models with ΔAICc ≤ 2.0 from the best model are shown in bold.

Variables	LogLikelihood	df	AICc	ΔAICc	AICc w
**Cav + Dist**	**-110.50**	**4**	**229.2**	**0.00**	**0.296**
**Cav**	**-111.92**	**3**	**229.9**	**0.76**	**0.202**
**Cav + Dist + Cav*Dist**	**-110.19**	**5**	**230.7**	**1.47**	**0.142**
Cav + Dist + BankVole	-110.47	5	231.2	2.03	0.107
Cav + BankVole	-111.92	4	232.0	2.84	0.072
Cav + Dist + BankVole + Cav*BankVole	-110.17	6	232.7	3.55	0.050
Cav + Dist + Bankvole + Cav*Dist	-110.17	6	232.5	3.55	0.050
Cav + Dist + BankVole + Dist*BankVole	-110.31	6	233.0	3.84	0.043
Cav + BankVole + Cav*BankVole	-111.53	5	233.3	4.15	0.037

Table S7 Model selection based on Akaike´s information criterion for small sample size (AICc) to determine which variables influenced the probability of predation of a boreal owl nest (*n* = 217) situated < 45 km from the microtine rodent trapping site in a year when microtine rodents were trapped (1977-78 and 1981-2018), with year as random effect (*n* = 35). Micr (range 0.00 – 2.77) denotes the *Microtus* vole spring trapping index (see text for details). Cav (range 1 – 15) denotes the time since the actual nest box was installed, with 1 denoting the first nesting season the box was available (see text). Dist (range -100 – 100) denotes the shortest distance from the nest box to the nearest edge between forest and an open habitat, with zero for boxes situated at the edge between forest and open habitat, a negative value for boxes situated within a forest stand, and a positive value for boxes situated in a tree in a clear-cut or a bog (see text for details). Models are sorted by their degree of support from the data, as indicated by the AICc weight (w). Models with ΔAICc ≤ 2.0 from the best model are shown in bold.

Variables	LogLikelihood	df	AICc	ΔAICc	AICc w
**Cav + Dist + Micr**	**-109.41**	**5**	**229.1**	**0.00**	**0.139**
**Cav + Dist**	**-110.50**	**4**	**229.2**	**0.08**	**0.134**
**Cav + Micr**	**-110.74**	**4**	**229.7**	**0.55**	**0.106**
**Cav**	**-111.92**	**3**	**229.9**	**0.84**	**0.091**
**Cav + Dist + Micr + Dist*Micr**	**-108.82**	**6**	**230.0**	**0.92**	**0.088**
**Cav + Dist + Micr + Cav*Dist + Cav*Micr + Dist*Micr + Cav*Dist*Micr**	**-105.64**	**9**	**230.1**	**1.03**	**0.083**
**Cav + Dist + Cav*Dist**	**-110.19**	**5**	**230.7**	**1.55**	**0.064**
**Cav + Dist + Micr + Cav*Micr**	**-109.14**	**6**	**230.7**	**1.58**	**0.063**
**Cav + Dist + Micr + Cav*Dist**	**-109.14**	**6**	**230.7**	**1.58**	**0.063**
**Cav + Micr + Cav*Micr**	**-110.35**	**5**	**231.0**	**1.88**	**0.054**
Cav + Dist + Micr + Cav*Micr + Dist*Micr	-108.56	7	231.7	2.55	0.039
Cav + Dist + Micr + Cav*Dist + Dist*Micr	-108.69	7	231.9	2.80	0.034
Cav + Dist + Micr + Cav*Dist + Cav*Micr	-108.93	7	232.4	3.28	0.027
Cav + Dist + Micr + Cav*Dist + Cav*Micr + Dist*Micr	-108.47	8	233.6	4.53	0.014

Table S8 Model selection based on Akaike´s information criterion for small sample size (AICc) to determine which variables influenced the probability of predation of a boreal owl nest (*n* = 217) situated < 45 km from the microtine rodent trapping site in a year when microtine rodents were trapped (1977-78 and 1981-2018), with year as random effect (*n* = 35). WL (range 0.00 – 2.86) denotes the wood lemming spring trapping index (see text for details). Cav (range 1 – 15) denotes the time since the actual nest box was installed, with 1 denoting the first nesting season the box was available (see text). Dist (range -100 – 100) denotes the shortest distance from the nest box to the nearest edge between forest and an open habitat, with zero for boxes situated at the edge between forest and open habitat, a negative value for boxes situated within a forest stand, and a positive value for boxes situated in a tree in a clear-cut or a bog (see text for details). Models are sorted by their degree of support from the data, as indicated by the AICc weight (w). Models with ΔAICc ≤ 2.0 from the best model are shown in bold.

Variables	LogLikelihood	df	AICc	ΔAICc	AICc w
**Cav + Dist**	**-110.50**	**4**	**229.2**	**0.00**	**0.146**
**Cav + Dist + WL + Cav*WL**	**-108.51**	**6**	**229.4**	**0.23**	**0.130**
**Cav + WL + Cav*WL**	**-109.58**	**5**	**229.4**	**0.26**	**0.128**
**Cav + Dist + WL + Cav*WL + Dist*WL**	**-107.58**	**7**	**229.7**	**0.50**	**0.113**
**Cav**	**-111.92**	**3**	**229.9**	**0.76**	**0.099**
**Cav + Dist + WL + Cav*Dist + Cav*WL + Dist*WL + Cav*Dist*WL**	**-105.69**	**9**	**230.2**	**1.06**	**0.086**
**Cav + Dist + Cav*Dist**	**-110.19**	**5**	**230.7**	**1.47**	**0.070**
Cav + Dist + WL + Cav*Dist + Cav*WL	-108.35	7	231.2	2.05	0.052
Cav + Dist + WL	-110.48	5	231.3	2.07	0.052
Cav + Dist + WL + Cav*Dist + Cav*WL + Dist*WL	-107.53	8	231.8	2.57	0.040
Cav + WL	-111.92	4	232.0	2.84	0.035
Cav + Dist + WL + Cav*Dist	-110.16	6	232.7	3.53	0.025
Cav + Dist + WL + Dist*WL	-110.21	6	232.8	3.63	0.024

Table S9 Parameter estimates from the highest-ranked model that included the actual microtine species in Tables S6-S8 for the probability of predation of a boreal owl nest situated < 45 km from the microtine rodent trapping site in a year when microtine rodents were trapped (n = 217), corrected for the random effect of year (n = 35). **a** Bank vole (Table S6). **b** *Microtus* voles (Table S7). **c** Wood lemming (Table S8).

Explanatory variable	Estimate ± SE	z	*P*
**a**			
Intercept	-1.291 ± 0.236	-5.477	< 0.0001
Bank vole abundance	0.056 ± 0.211	0.264	0.79
Cavity age	0.710 ± 0.172	4.138	< 0.0001
Distance to forest edge	-0.307 ± 0.187	-1.644	0.10
**b**			
Intercept	-1.230 ± 0.208	-5.922	< 0.0001
Microtus voles abundance	0.317 ± 0.203	1.560	0.12
Cavity age	0.653 ± 0.174	3.762	0.0002
Distance to forest edge	-0.288 ± 0.184	-1.570	0.12
**c**			
Intercept	-1.271 ± 0.232	-5.486	< 0.0001
Wood lemming abundance	0.076 ± 0.192	0.395	0.69
Cavity age	0.793 ± 0.180	4.412	< 0.0001
Distance to forest edge	-0.263 ± 0.185	-1.424	0.15
Cavity age * Wood lemming abundance	-0.333 ± 0.176	-1.887	0.059

Generalized linear mixed-effect models with log link function, binomial distribution, and Adaptive Gause-Hermite quadrature approximation to the likelihood. Explanatory variables are standardized.

Table S10 Model selection based on Akaike´s information criterion (AIC) to determine which variables influenced the probability of predation of a boreal owl nest situated < 45 km from the microtine rodent trapping site in a year when microtine rodents had also been trapped in the previous year (*n* = 187), with year (*n* = 33) as random variable. MicrotineCh (range -3.53 – 5.03) denotes the change in the microtine rodent trapping index from the previous spring to the current spring (see text for details). Cav (range 1 – 15) denotes the time since the actual nest box was installed (see text for details). Dist (range -100 – 100) denotes the shortest distance from the nest box to the nearest edge between forest and an open habitat (see text for details). In all models Year in included as a random variable. Models are sorted by their degree of support from the data, as indicated by the AICc weight (w). Models with ΔAIC ≤ 2.0 from the best model are shown in bold.

Variables	LogLikelihood	df	AICc	ΔAIC	AIC w
**Cav**	**-89.63**	**3**	**185.4**	**0.00**	**0.442**
**Cav + Dist**	**-89.37**	**4**	**187.0**	**1.58**	**0.200**
Cav + MicrotineCh	-89.63	4	187.5	2.09	0.156
Cav + Dist + Cav*Dist	-89.33	5	189.0	3.60	0.073
Cav + Dist + MicrotineCh	-89.37	5	189.1	3.69	0.070
Cav + MicrotineCh + Cav*MicrotineCh	-89.54	5	189.4	4.02	0.059

Table S11 Model selection based on Akaike´s information criterion for small sample size (AICc) to determine which variables influenced the probability of predation of a boreal owl nest (*n* = 187) situated < 45 km from the microtine rodent trapping site in a year when microtine rodents had been trapped the previous year (1978 and 1982-2018), with year (*n* = 33) as random variable. BankVoleCh (range -1.63 – 2.64) denotes the change in the bank vole trapping index from the previous spring to the current spring (see text for details). Cav (range 1 – 15) denotes the time since the actual nest box was installed, with 1 denoting the first nesting season the box was available (see text). Dist (range -100 – 100) denotes the shortest distance from the nest box to the nearest edge between forest and an open habitat, with zero for boxes situated at the edge between forest and open habitat, a negative value for boxes situated within a forest stand, and a positive value for boxes situated in a tree in a clear-cut or a bog (see text for details). In all models Year in included as a random variable. Models are sorted by their degree of support from the data, as indicated by the AICc weight (w). Models with ΔAICc ≤ 2.0 from the best model are shown in bold.

Variables	LogLikelihood	df	AICc	ΔAICc	AICc w
**Cav**	**-89.63**	**3**	**185.4**	**0.00**	**0.403**
**Cav + Dist**	**-89.37**	**4**	**187.0**	**1.58**	**0.182**
Cav + BankVoleCh	-89.60	4	187.4	2.04	0.145
Cav + BankVoleCh + Cav*BankVoleCh	-88.95	5	188.2	2.86	0.097
Cav + Dist + Cav*Dist	-89.33	5	189.0	3.60	0.067
Cav + Dist + BankVoleCh	-89.34	5	189.0	3.62	0.066
Cav + Dist + BankVoleCh + Cav*BankVoleCh	-88.77	6	190.0	4.62	0.040

Table S12 Model selection based on Akaike´s information criterion for small sample size (AICc) to determine which variables influenced the probability of predation of a boreal owl nest (*n* = 187) situated < 45 km from the microtine rodent trapping site in a year when microtine rodents had been trapped the previous year (1978 and 1982-2018), with year (*n* = 33) as random variable. MicVolesCh (range -1.81 – 1.74) denotes the change in the *Microtus* vole trapping index from the previous spring to the current spring (see text for details). Cav (range 1 – 15) denotes the time since the actual nest box was installed, with 1 denoting the first nesting season the box was available (see text). Dist (range -100 – 100) denotes the shortest distance from the nest box to the nearest edge between forest and an open habitat, with zero for boxes situated at the edge between forest and open habitat, a negative value for boxes situated within a forest stand, and a positive value for boxes situated in a tree in a clear-cut or a bog (see text for details). In all models Year in included as a random variable. Models are sorted by their degree of support from the data, as indicated by the AICc weight (w). Models with ΔAICc ≤ 2.0 from the best model are shown in bold.

Variables	LogLikelihood	df	AICc	ΔAICc	AICc w
**Cav**	**-89.63**	**3**	**185.4**	**0.00**	**0.415**
**Cav + Dist**	**-89.37**	**4**	**187.0**	**1.58**	**0.188**
**Cav + MicVolesCh**	**-89.41**	**4**	**187.0**	**1.66**	**0.181**
Cav + Dist + MicVolesCh	-89.17	5	188.7	3.30	0.080
Cav + Dist + Cav*Dist	-89.33	5	189.0	3.60	0.069
Cav + MicVolesCh + Cav*MicVolesCh	-89.35	5	189.0	3.64	0.067

Table S13 Model selection based on Akaike´s information criterion for small sample size (AICc) to determine which variables influenced the probability of predation of a boreal owl nest (*n* = 187) situated < 45 km from the microtine rodent trapping site in a year when microtine rodents had been trapped the previous year (1978 and 1982-2018), with year (*n* = 33) as random variable. WlCh (range -0.45 – 2.68) denotes the change in the wood lemming trapping index from the previous spring to the current spring (see text for details). Cav (range 1 – 15) denotes the time since the actual nest box was installed, with 1 denoting the first nesting season the box was available (see text). Dist (range -100 – 100) denotes the shortest distance from the nest box to the nearest edge between forest and an open habitat, with zero for boxes situated at the edge between forest and open habitat, a negative value for boxes situated within a forest stand, and a positive value for boxes situated in a tree in a clear-cut or a bog (see text for details). In all models Year in included as a random variable. Models are sorted by their degree of support from the data, as indicated by the AICc weight (w). Models with ΔAICc ≤ 2.0 from the best model are shown in bold.

Variables	LogLikelihood	df	AICc	ΔAICc	AICc w
**Cav**	**-89.63**	**3**	**185.4**	**0.00**	**0.284**
**Cav + WlCh + Cav*WlCh**	**-88.19**	**5**	**186.7**	**1.33**	**0.146**
**Cav + Dist**	**-89.37**	**4**	**187.0**	**1.58**	**0.129**
**Cav + Dist + WlCh + Cav*Dist + Cav*WlCh + Dist*WlCh + Cav*Dist*WlCh**	**-84.12**	**9**	**187.3**	**1.88**	**0.111**
**Cav + WlCh**	**-89.56**	**4**	**187.3**	**1.95**	**0.107**
Cav + Dist + WlCh + Cav*WlCh	-88.02	6	188.5	3.12	0.060
Cav + Dist + WlCh	-89.26	5	188.9	3.47	0.050
Cav + Dist + Cav*Dist	-89.33	5	189.0	3.60	0.047
Cav + Dist + WlCh + Cav*WlCh + Dist*WlCh	-87.28	7	189.2	3.81	0.042
Cav + Dist + WlCh + Dist*WlCh	-88.95	6	190.4	4.99	0.023

Table S14 Parameter estimates from the highest-ranked models that included the microtine species in Tables S11-S13, and from the most parsimonious models that included the microtine species and the distance to forest edge in Tables S11-S13, for the probability of predation of a boreal owl nest (*n* = 187) situated < 45 km from the microtine rodent trapping site in a year when microtine rodents had been trapped the previous year, corrected for the random effect of year (*n* = 33). **a** The highest-ranked model that included bank vole (Table S11). **b** The most parsimonious model that included bank vole and distance to forest edge (Table S11). **c** The highest-ranked model that included *Microtus* voles (Table S12). **d** The most parsimonious model that included *Microtus* voles and distance to forest edge (Table S12). **e** The highest-ranked model that included wood lemming (Table S13). **f** The most parsimonious model that included wood lemming and distance to forest edge (Table S13).

Explanatory variable	Estimate ± SE	z	*P*
**a**			
Intercept	-1.439 ± 0.192	-7.490	< 0.0001
Bank vole change	0.040 ± 0.181	0.223	0.82
Cavity age	0.516 ± 0.165	3.120	0.0018
**b**			
Intercept	-1.442 ± 0.193	-7.489	< 0.0001
Bank vole change	0.050 ± 0.182	0.277	0.78
Cavity age	0.511 ± 0.166	3.083	0.0021
Distance to forest edge	-0.139 ± 0.194	-0.717	0.47
**c**			
Intercept	-1.444 ± 0.193	-7.483	< 0.0001
Microtus voles change	-0.116 ± 0.176	-0.657	0.51
Cavity age	0.523 ± 0.166	3.147	0.0017
**d**			
Intercept	-1.447 ± 0.193	-7.483	< 0.0001
Microtus vole change	-0.113 ± 0.178	-0.636	0.53
Cavity age	0.518 ± 0.167	3.108	0.0019
Distance to forest edge	-0.130 ± 0.192	-0.679	0.50
**e**			
Intercept	-1.425 ± 0.195	-7.322	< 0.0001
Wood lemming change	0.112 ± 0.172	0.652	0.51
Cavity age	0.577 ± 0.175	3.305	0.0009
Cavity age * Wood lemming change	-0.326 ± 0.210	-1.550	0.12
**f**			
Intercept	-1.428 ± 0.195	-7.325	< 0.0001
Wood lemming change	0.122 ± 0.174	0.699	0.48
Cavity age	0.570 ± 0.175	3.250	0.0012
Distance to forest edge	-0.113 ± 0.192	-0.588	0.56
Cavity age * Wood lemming change	-0.315 ± 0.212	-1.488	0.14

Generalized linear mixed-effect models with log link function, binomial distribution, and Adaptive Gause-Hermite quadrature approximation to the likelihood. Explanatory variables are standardized.

Table S15 Model selection based on Akaike´s information criterion for small sample size (AICc) to determine which variables influenced the probability of predation of a boreal owl nest (*n* = 214) situated < 45 km from the microtine rodent trapping site in a year when microtine rodents were trapped (1977-78 and 1981-2018), with year as random effect (*n* = 33). Clutch (range 3-10) denotes the clutch size of the actual nest or its nearest neighbor in the same year if the actual nest was predated before the first nest check (see text for details). Cav (range 1 – 15) denotes the time since the actual nest box was installed, with 1 denoting the first nesting season the box was available (see text). Dist (range -100 – 100) denotes the shortest distance from the nest box to the nearest edge between forest and an open habitat, with zero for boxes situated at the edge between forest and open habitat, a negative value for boxes situated within a forest stand, and a positive value for boxes situated in a tree in a clear-cut or a bog (see text for details). Models are sorted by their degree of support from the data, as indicated by the AICc weight (w). Models with ΔAICc ≤ 2.0 from the best model are shown in bold.

Variables	LogLikelihood	df	AICc	ΔAICc	AICc w
**Cav + Dist**	**-106.72**	**4**	**221.6**	**0.00**	**0.245**
**Cav**	**-108.07**	**3**	**222.3**	**0.62**	**0.180**
**Cav + Dist + Cav*Dist**	**-106.39**	**5**	**223.1**	**1.44**	**0.119**
**Cav + Dist + Clutch**	**-106.45**	**5**	**223.2**	**1.56**	**0.112**
Cav + Clutch	-107.85	4	223.9	2.26	0.079
Cav + Dist + Clutch + Dist*Clutch	-106.02	6	224.4	2.81	0.060
Cav + Dist + Clutch + Cav*Dist	-106.11	6	224.6	3.00	0.055
Cav + Dist + Clutch + Cav*Clutch	-106.30	6	225.0	3.36	0.046
Cav + Clutch + Cav*Clutch	-107.66	5	225.6	3.98	0.034
Cav + Dist + Clutch + Cav*Dist + Dist*Clutch	-105.85	7	226.2	4.60	0.025
Cav + Dist + Clutch + Cav*Clutch + Dist*Clutch	-105.91	7	226.4	4.73	0.023
Cav + Dist + Clutch + Cav*Clutch + Cav*Dist	-105.99	7	226.5	4.88	0.021

Table S16 Parameter estimates from the highest-ranked model that included clutch size in Table S15 for the probability of predation of a boreal owl nest (*n* = 214) situated < 45 km from the microtine rodent trapping site in the years when microtine rodent were trapped, corrected for the random effect of year (*n* = 33).

Explanatory variable	Estimate ± SE	z	*P*
Intercept	-1.383 ± 0.243	-5.696	< 0.0001
Boreal owl clutch size	0.135 ± 0.184	0.733	0.46
Cavity age	0.699 ± 0.174	4.025	< 0.0001
Distance to forest edge	-0.303 ± 0.187	-1.616	0.11

Generalized linear mixed-effect models with log link function, binomial distribution, and Adaptive Gause-Hermite quadrature approximation to the likelihood. Explanatory variables are standardized.

Table S17 Model selection based on Akaike´s information criterion for small sample size (AICc) to determine which variables influenced the probability of predation of a boreal owl nest (n = 337), with year as random effect (n = 40). Clutch (range 2 – 10) denotes the clutch size of the actual boreal owl nest, or the clutch size of the nearest recorded neigbor nest in the same year in case the actual nest was predated prior to the first nest check, and is a proxy for the microtine rodent abundance at the actual nest (see text for details). Cav (range 1 – 15) denotes the time since the actual nest box was installed, with 1 denoting the first nesting season the box was available (see text). Dist (range -100 – 100) denotes the shortest distance from the nest box to the nearest edge between forest and an open habitat, with zero for boxes situated at the edge between forest and open habitat, a negative value for boxes situated within a forest stand, and a positive value for boxes situated in a tree in a clear-cut or a bog (see text for details). Models are sorted by their degree of support from the data, as indicated by the AICc weight (w). Models with ΔAICc ≤ 2.0 from the best model are shown in bold.

Variables	LogLikelihood	df	AICc	ΔAICc	AICc w
**Cav + Dist**	**-167.08**	**4**	**342.3**	**0.00**	**0.380**
**Cav + Dist + Cav*Dist**	**-166.68**	**5**	**343.5**	**1.27**	**0.201**
**Cav + Dist + Clutch**	**-167.01**	**5**	**344.2**	**1.93**	**0.144**
Cav + Dist + Clutch + Cav*Dist	-166.59	6	345.4	3.16	0.078
Cav + Dist + Clutch + Dist*Clutch	-166.80	6	345.9	3.59	0.063
Cav + Dist + Clutch + Cav*Clutch	-166.97	6	346.2	3.92	0.053
Cav	-170.19	3	346.5	4.18	0.047
Cav + Dist + Clutch + Cav*Dist + Dist*Clutch	-166.97	7	346.2	4.86	0.033

Table S18 Parameter estimates from the highest-ranked model that included clutch size in Table S17 for the probability of predation of a boreal owl nest (*n* = 337), corrected for the random effect of year (*n* = 40).

Explanatory variable	Estimate ± SE	z	*P*
Intercept	-1.355 ± 0.140	-9.709	< 0.0001
Boreal owl clutch size	0.049 ± 0.136	0.358	0.72
Cavity age	0.410 ± 0.122	3.353	0.0008
Distance to forest edge	- 0.330 ± 0.137	-2.418	0.016

Generalized linear mixed model with log link function, binomial distribution, and Adaptive Gause-Hermite quadrature approximation to the likelihood. Continuous explanatory variables are standardized.

Table S19 Model selection based on Akaike´s information criterion for small sample size (AICc) to determine which variables influenced the probability of predation of a boreal owl nest (*n* = 77) in a nest box the first season after it was installed (cavity age = 1) situated < 45 km from the microtine rodent trapping site in a year when microtine rodents were trapped (1977-78 and 1981-2018), with year as random effect (*n* = 23). MicRod (range 0.18 – 4.02) denotes the microtine rodent spring trapping index (see text for details). Dist (range -100 – 100) denotes the shortest distance from the nest box to the nearest edge between forest and an open habitat, with zero for boxes situated at the edge between forest and open habitat, a negative value for boxes situated within a forest stand, and a positive value for boxes situated in a tree in a clear-cut or on a bog were as (see text for details). Models are sorted by their degree of support from the data, as indicated by the AICc weight (w). Models with ΔAICc ≤ 2.0 from the best model are shown in bold.

Variables	LogLikelihood	df	AICc	ΔAICc	AICc w
**Intercept**	**-25.68**	**2**	**55.5**	**0.00**	**0.472**
**MicRod**	**-25.21**	**3**	**56.8**	**1.22**	**0.256**
Dist	-25.61	3	57.5	2.01	0.173
Dist + MicRod	-25.06	4	58.7	3.14	0.098

Table S20 Model selection based on Akaike´s information criterion for small sample size (AICc) to determine which variables influenced the probability of predation of a boreal owl nest (*n* = 71) in a nest box the first season after it was installed (cavity age = 1) situated < 45 km from the microtine rodent trapping site in a year when microtine rodents had been trapped the previous year (1978 and 1982-2018), with year (*n* = 21) as random variable. MicrotineCh (range -2.07 – 1.34) denotes the change in the microtine rodent trapping index from the previous spring to the current spring (see text for details). DistEdge (range -100 – 100) denotes the shortest distance from the nest box to the nearest edge between forest and an open habitat, with zero for boxes situated at the edge between forest and open habitat, a negative value for boxes situated within a forest stand, and a positive value for boxes situated in a tree in a clear-cut or on a bog were as (see text for details). In all models Year in included as a random variable. Explanatory variables are standardized. Models are sorted by their degree of support from the data, as indicated by the AICc weight (w). Models with ΔAICc ≤ 2.0 from the best model are shown in bold.

Variables	LogLikelihood	df	AICc	ΔAICc	AICc w
**Intercept**	**-25.00**	**2**	**54.2**	**0.00**	**0.546**
**Dist**	**-24.91**	**3**	**56.2**	**2.00**	**0.201**
MicrotineCh	-24.99	3	56.3	2.16	0.186
Dist + MicrotineCh	-24.88	4	58.4	4.20	0.067

Table S21 Model selection based on Akaike´s information criterion for small sample size (AICc) to determine which variables influenced the probability of predation of a boreal owl nest (*n* = 151) in a nest box the first season after it was installed (cavity age = 1), with year as random effect (*n* = 31). Clutch (range 2-8) denotes the clutch size of the actual nest or its nearest neighbor in the same year if the actual nest was predated before the first nest check (see text for details). Dist (range -100 – 100) denotes the shortest distance from the nest box to the nearest edge between forest and an open habitat, with zero for boxes situated at the edge between forest and open habitat, a negative value for boxes situated within a forest stand, and a positive value for boxes situated in a tree in a clear-cut or a bog (see text for details). Models are sorted by their degree of support from the data, as indicated by the AICc weight (w). Models with ΔAICc ≤ 2.0 from the best model are shown in bold.

Variables	LogLikelihood	df	AICc	ΔAICc	AICc w
**Intercept**	**-57.14**	**2**	**118.4**	**0.00**	**0.420**
**Dist**	**-56.49**	**3**	**119.1**	**0.78**	**0.284**
**Clutch**	**-57.07**	**3**	**120.3**	**1.96**	**0.158**
Clutch + Dist	-56.47	4	121.2	2.86	0.101
Dist + Clutch + Dist*Clutch	-56.40	5	123.2	4.87	0.037

Table S22 Parameter estimates from the highest-ranked models that included microtine rodent abundance alone (**a**) or in combination with distance to forest edge (**b**) in Table S19 for the probability of predation of a boreal owl nest (*n* = 77) in a nest box the first season after it was installed (cavity age = 1), corrected for the random effect of year (*n* = 23).

Explanatory variable	Estimate ± SE	z	*P*
**a**			
Intercept	-2.217 ± 0.398	-5.572	< 0.0001
Microtine rodent abundance	-0.421 ± 0.471	-0.893	0.37
**b**			
Intercept	-2.236 ± 0.404	-5.533	< 0.0001
Microtine rodent abundance	-0.457 ± 0.476	-0.961	0.34
Distance to forest edge	-0.224 ± 0.390	-0.575	0.57

Generalized linear mixed model with log link function, binomial distribution, and Adaptive Gause-Hermite quadrature approximation to the likelihood. Continuous explanatory variables are standardized.

Table S23 Parameter estimates from the models that included change in microtine rodent abundance alone (**a**) or in combination with distance to forest edge (**b**) in Table S20 for the probability of predation of a boreal owl nest (*n* = 71) in a nest box the first season after it was installed (cavity age = 1), corrected for the random effect of year (*n* = 21).

Explanatory variable	Estimate ± SE	z	*P*
**a**			
Intercept	-2.065 ± 0.376	-5.495	< 0.0001
Microtine rodent change	0.057 ± 0.387	0.148	0.88
**b**			
Intercept	-2.076 ± 0.379	-5.474	< 0.0001
Microtine rodent change	0.085 ± 0.395	0.214	0.83
Distance to forest edge	-0.170 ± 0.365	-0.464	0.64

Generalized linear mixed model with log link function, binomial distribution, and Adaptive Gause-Hermite quadrature approximation to the likelihood. Continuous explanatory variables are standardized.

Table S24 Parameter estimates from the models that included boreal owl clutch size alone (**a**) or in combination with distance to forest edge (**b**) in Table S21 for the probability of predation of a boreal owl nest (*n* = 151) in a nest box the first season after it was installed (cavity age = 1), corrected for the random effect of year (*n* = 31).

Explanatory variable	Estimate ± SE	z	*P*
**a**			
Intercept	-1.941 ± 0.241	-7.891	< 0.0001
Boreal owl clutch size	0.087 ± 0.246	0.356	0.72
**b**			
Intercept	-1.964 ± 0.250	-7.847	< 0.0001
Boreal owl clutch size	0.047 ± 0.247	0.192	0.85
Distance to forest edge	-0.249 ± 0.219	-1.137	0.26

Generalized linear mixed model with log link function, binomial distribution, and Adaptive Gause-Hermite quadrature approximation to the likelihood. Continuous explanatory variables are standardized.
