# Supplementary material for: Predation of boreal owl nests by pine martens in the boreal forest does not vary as predicted by the alternative prey hypothesis
Source: Oecologia. 2022 Mar 19;198(4):995–1009. doi: 10.1007/s00442-022-05149-0 (PMC9056444; doi:10.1007/s00442-022-05149-0)
Supplement: Supplementary file 2 — Supplementary file2 (PDF 619 KB) [file 442_2022_5149_MOESM2_ESM.pdf]

Electronic Supplementary Material (ESM 2)

Sonerud GA (2022) Predation of boreal owl nests by pine martens in the boreal forest does not vary as predicted by the alternative prey hypothesis

Figures S1 – S9

Oecologia

Geir A. Sonerud

Faculty of Environmental Sciences and Natural Resource Management

Norwegian University of Life Sciences

P. O. Box 5003

NO-1432 Ås

Norway

E-mail: geir.sonerud@nmbu.no

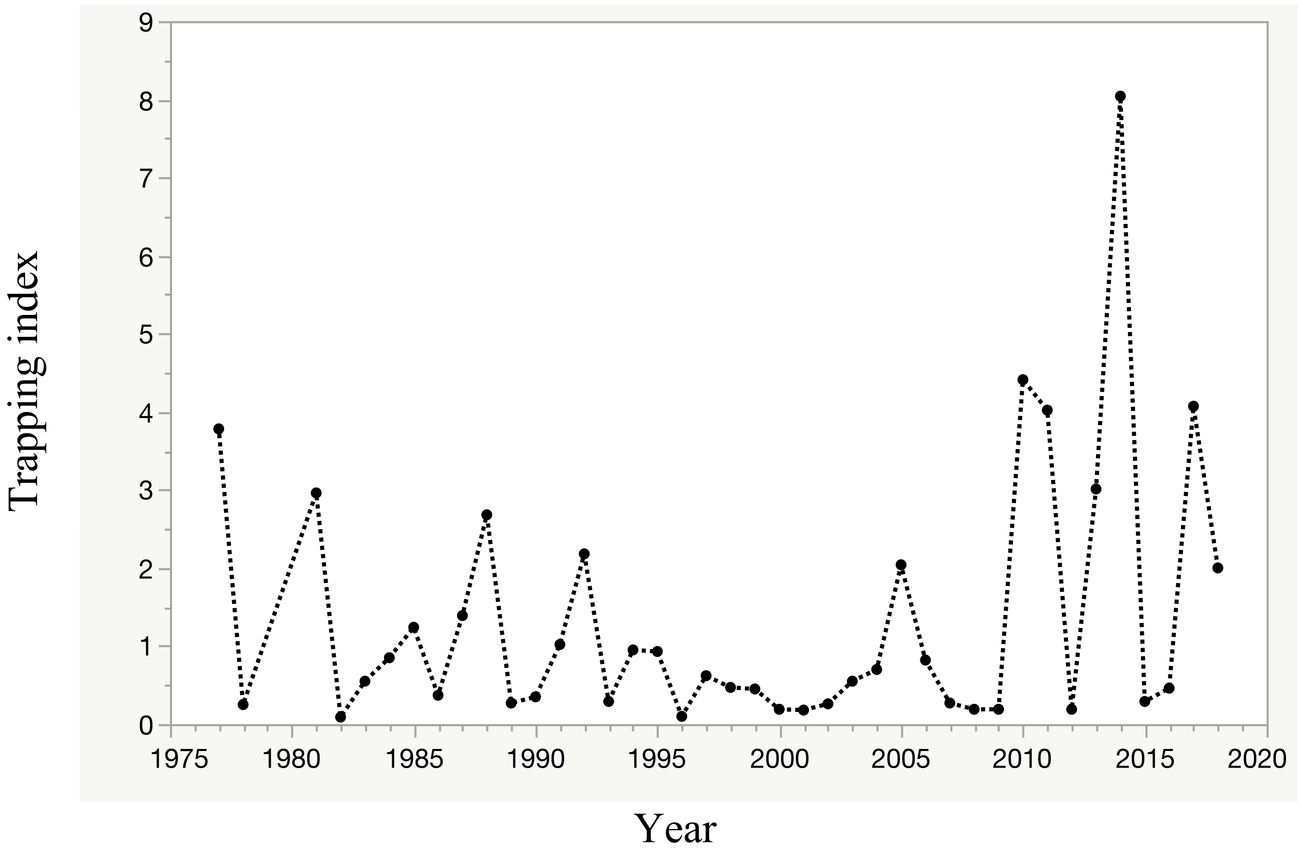

Fig. S1 Microtine rodent spring snap trapping index (numbers trapped per 100 trap nights for bank vole (*Myodes glareolus*), field vole (*Microtus agrestis*), tundra vole (*Microtus oeconomus*) and wood lemming (*Myopus schisticolor*) pooled) during 1977-78 and 1981-2018 in the boreal forest 550-600 m a.s.l. at 60°56´N, 11°08´E in southeast Norway (see Fig. 1).

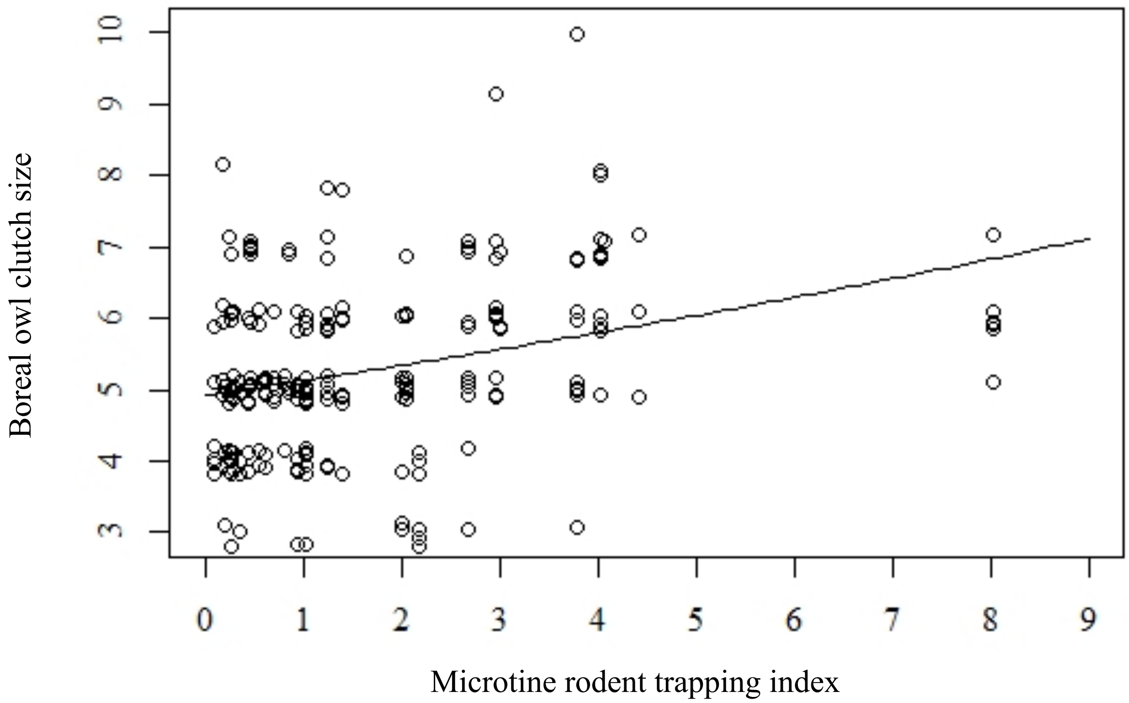

Fig. S2 The clutch size of a boreal owl nest in a nest box situated < 45 km from the microtine rodent trapping site as function of the microtine rodent trapping index in spring in the years when microtine rodents were trapped (1977-78 and 1981-2018) (n = 223, Poisson distribution, slope = 0.041 ± 0.016, z = 2.512, P = 0.012). Overlapping data points are spread by using the jitter function in R.

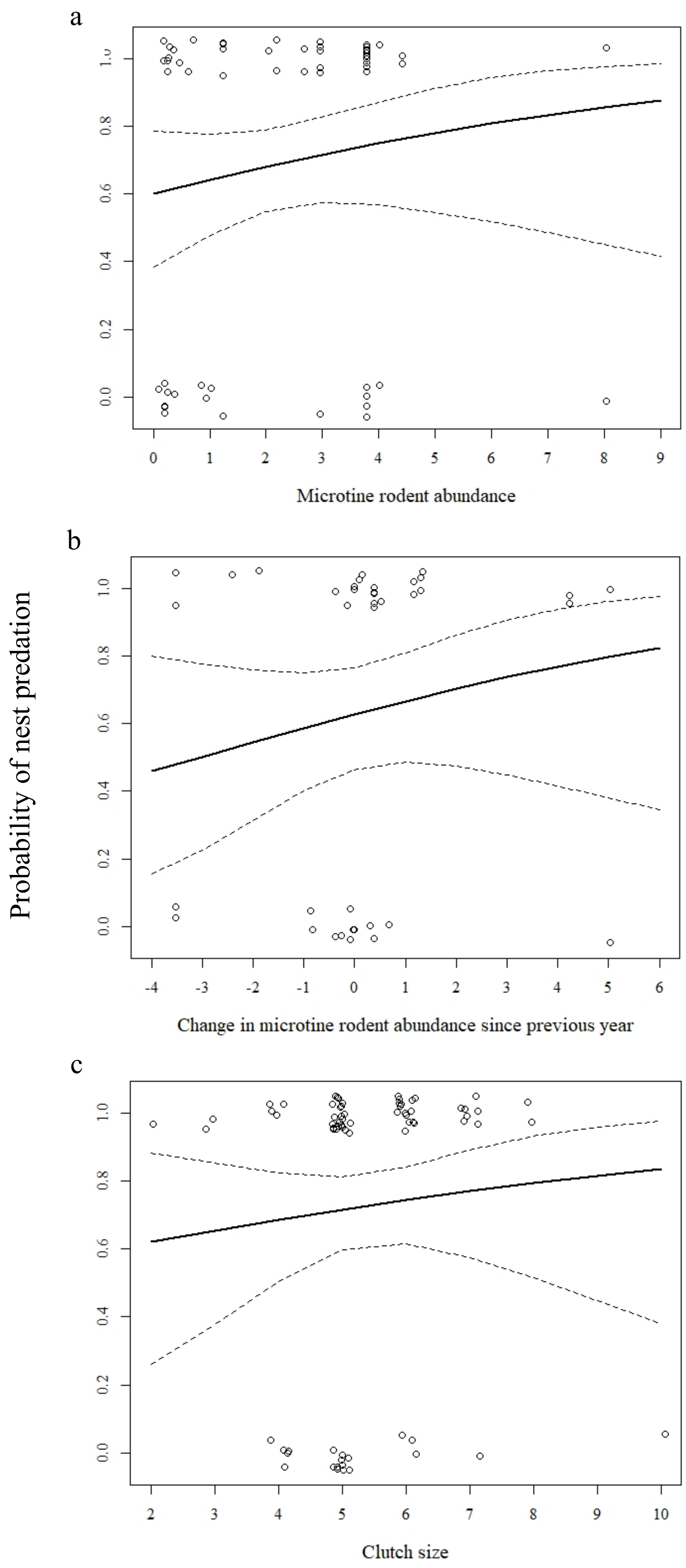
a
b
c
Probability of nest predation
Microtine rodent abundance
Change in microtine rodent abundance since previous year
Clutch size

Previous page:

Fig. S3 The probability that the predator of a predated boreal owl nest was scored as pine marten rather than as an unidentified predator as a function of a) the microtine rodent trapping index in spring (n = 58, slope = 0.172 ± 0.165, z = 1.042, P = 0.30), b) the change in microtine rodent abundance from the previous spring to the current spring (n = 38, slope = 0.171 ± 0.178, z = 0.963, P = 0.34), and c) the size of the actual boreal owl clutch predated or the size of the clutch of the nearest neighbor, taken as a proxy for the microtine rodent abundance (n = 73, slope = 0.142 ± 0.224, z = 0.635, P = 0.53). Data from one nest per nest box. Overlapping data points are spread by using the jitter function in R.

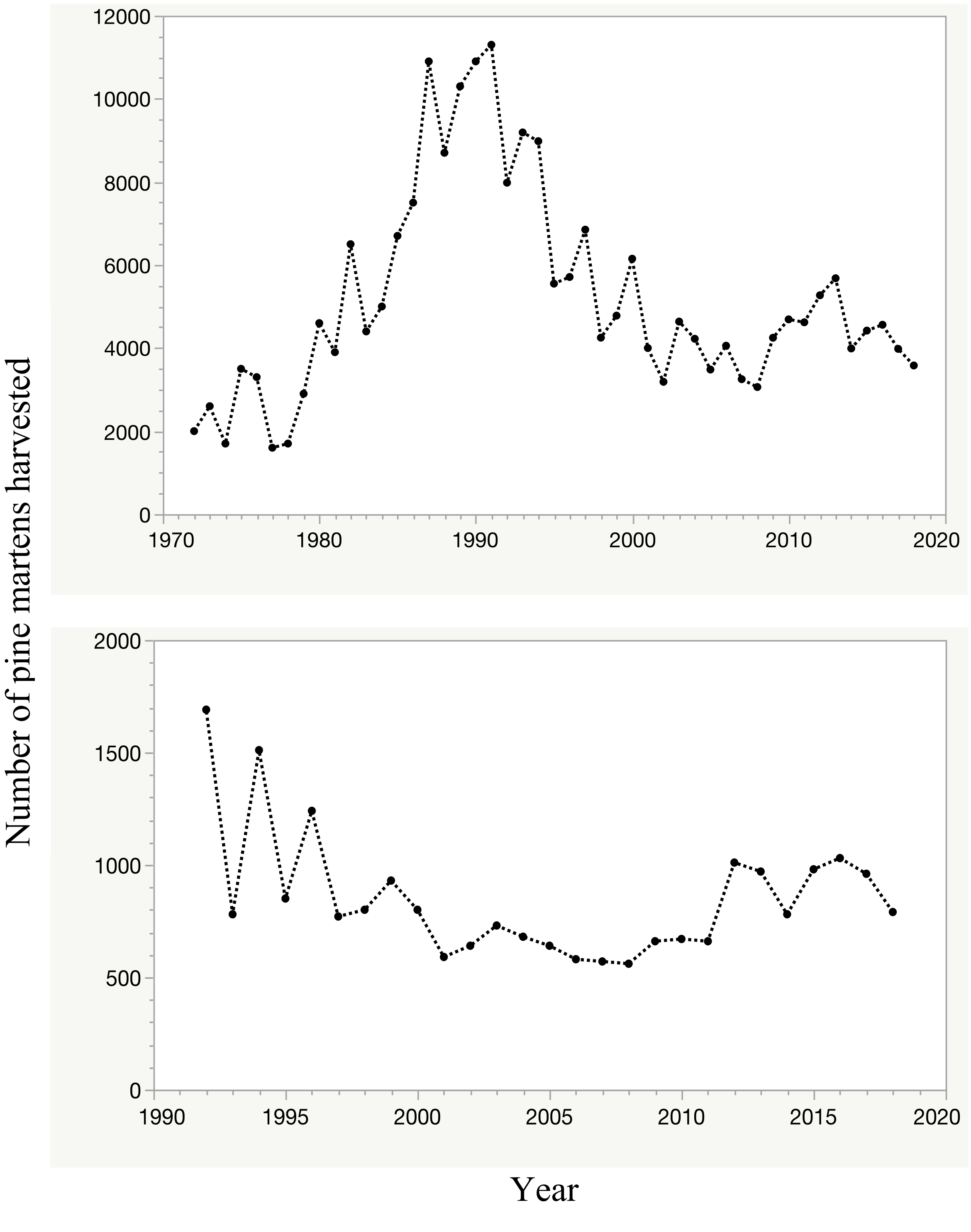

Fig. S4 Recorded annual number of pine martens harvested in Norway (1972-2018, upper panel) and in Hedmark and Oppland counties (1992-2018, lower panel). The number assigned to year N is the hunting bag of pine marten during the trapping season from November in year N-1 to March in year N. Data extracted from Statistics Norway (2020).

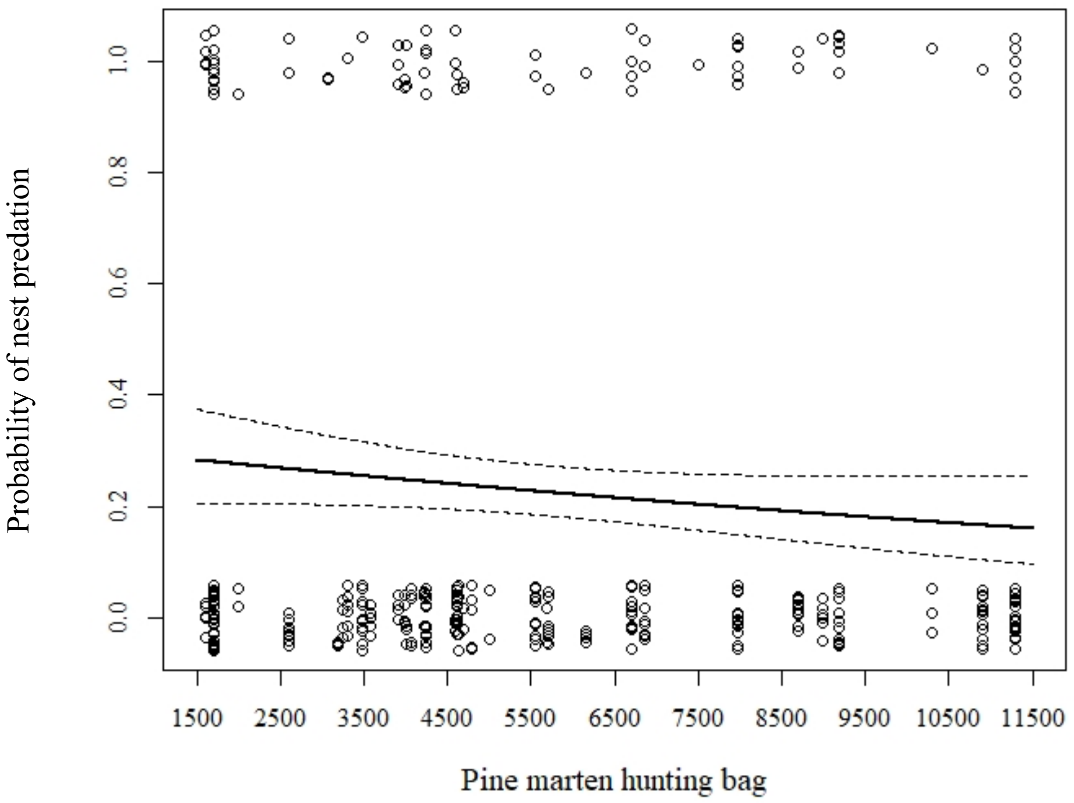

Fig. S5 The probability of predation of a boreal owl nest as function of the pine marten hunting bag in Norway, with data from one nest per nest box (*n* = 336, slope = (-0.731 ± 0.437) x 10^{-4}, z = - 1.674, *P* = 0.094). Overlapping data points are spread by using the jitter function in R.

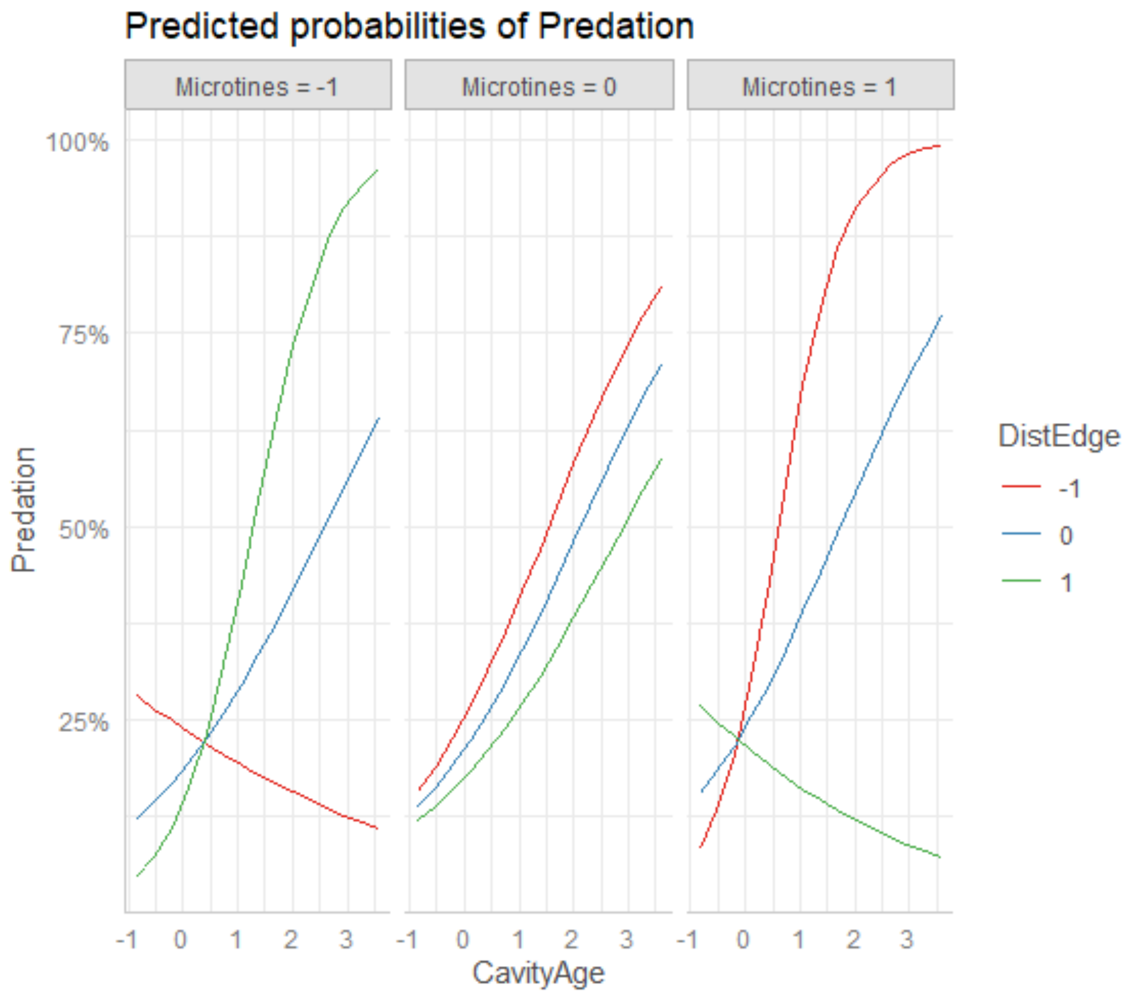

Fig. S6 The probability of predation of a boreal owl nest as affected by the triple interaction between cavity age, distance to edge between open habitat and habitat with forest cover, and microtine index, in the full model shown in Table 1c. The three levels for distance to edge (DistEdge) and for microtine index (Microtines) are based on standardized variables. For distance to edge, -1 corresponds to 20 m into forest from the edge, 0 corresponds to 6 m into open habitat from the edge, and 1 corresponds to 33 m into open habitat from the edge. For microtine index, -1 corresponds to 0.2, 0 corresponds to 2, and 1 corresponds to 4.

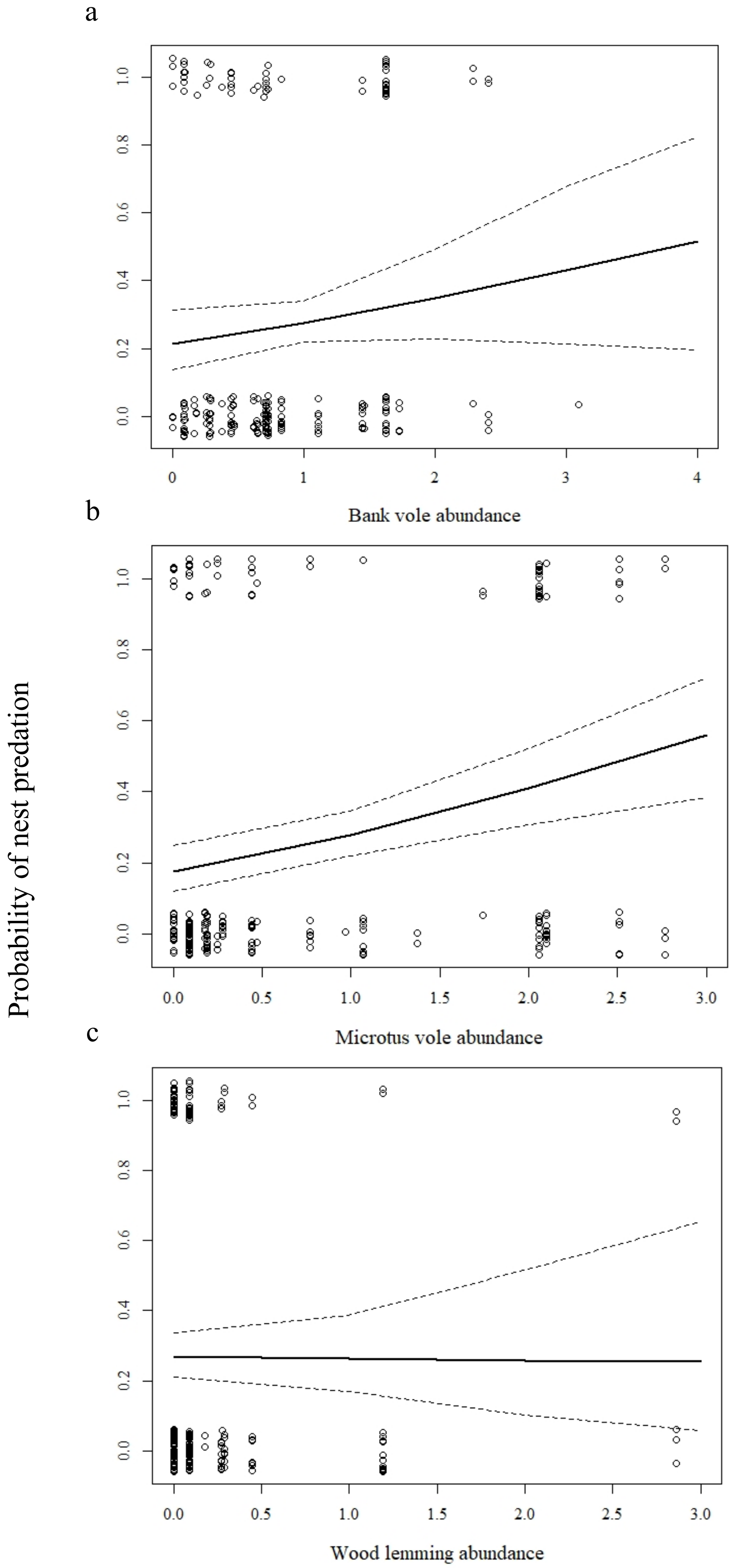

a
Bank vole abundance
b
Microtus vole abundance
c
Wood lemming abundance
Probability of nest predation

Previous page:

Fig. S7 The probability of predation of a boreal owl nest as function of the spring trapping index of the microtine rodent species groups separately, with data from one nest per nest box. a) Bank vole (n = 217, slope = 0.344 ± 0.239, z = 1.436, P = 0.15). b) *Microtus* voles (n = 217, slope = 0.595 ± 0.164, z = 3.618, P = 0.0003). c) Wood lemming (n = 217, slope = -0.026 ± 0.310, z = -0.085, P = 0.93). Overlapping data points are spread by using the jitter function in R.

Next page:

Fig. S8 The probability of predation of a boreal owl nest as function of the change in spring trapping index of the microtine rodents from the previous year, with data from one nest per nest box. a) Microtine rodents (n = 187, slope = 0.014 ± 0.115, z = 0.129, P = 0.90). b) Bank vole (n = 217, slope = 0.008 ± 0.212, z = 0.039, P = 0.97). c) *Microtus* voles (n = 217, slope = -0.125 ± 0.274, z = -0.455, P = 0.65). d) Wood lemming (n = 217, slope = 0.249 ± 0.323, z = 0.769, P = 0.44). Overlapping data points are spread by using the jitter function in R.

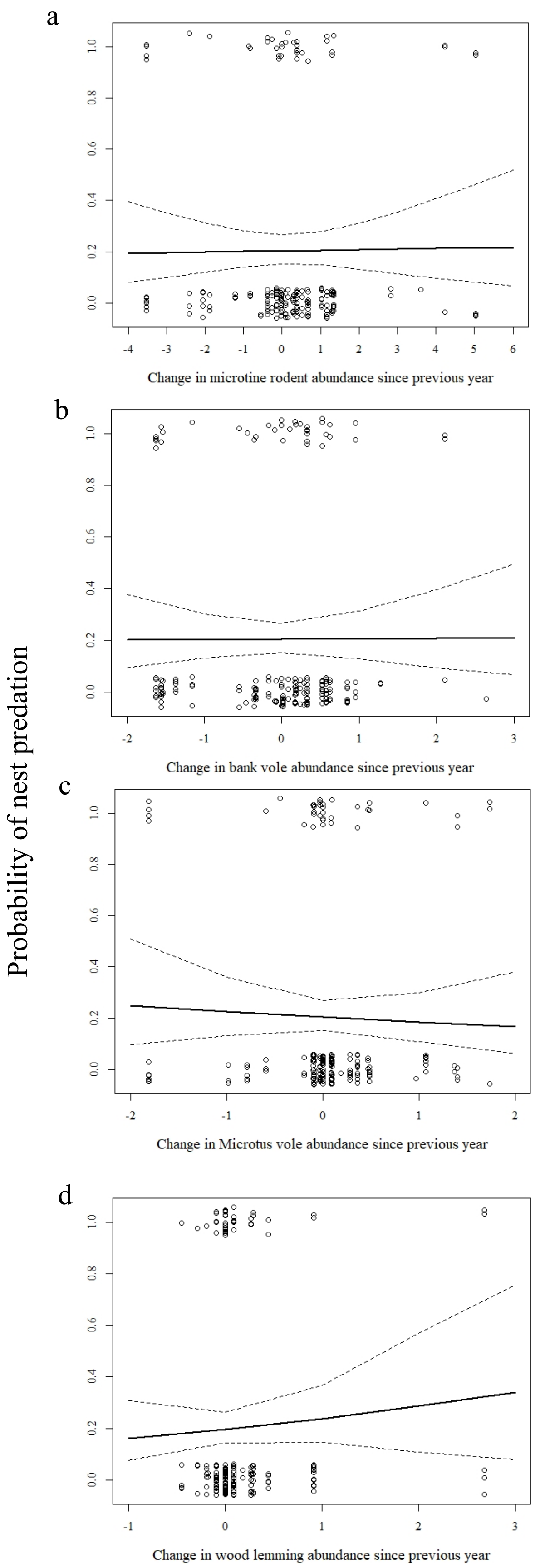
a
Change in microtine rodent abundance since previous year
b
Change in bank vole abundance since previous year
c
Change in Microtus vole abundance since previous year
d
Change in wood lemming abundance since previous year
Probability of nest predation

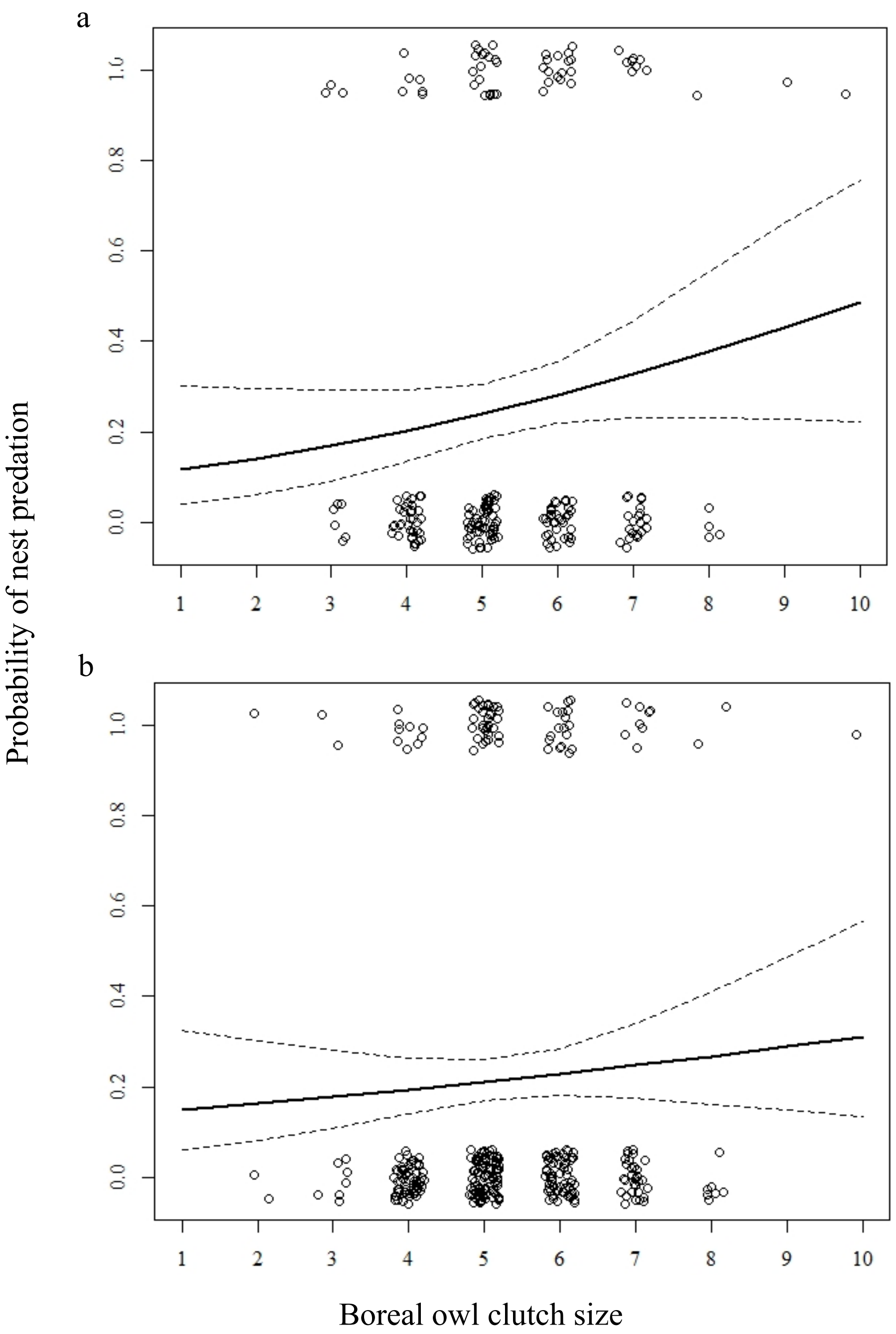

Fig. S9 The probability of predation of a boreal owl nest as function of the clutch size as proxy of the microtine rodent abundance, either the clutch size of the actual nest or the clutch size of the nearest recorded neighbor nest in the same year in case the actual nest was predated prior to the first nest check, with data from one nest per nest box. a) Nest boxes situated < 45 km from the microtine rodent trapping site in the years when microtine rodents were trapped (1977-78 and 1981-2018) (n = 214, slope = 0.219 ± 0.130, z = 1.682, P = 0.093). b) All nest boxes in all study years (n = 337, slope = 0.104 ± 0.114, z = 0.914, P = 0.36). Overlapping data points are spread by using the jitter function in R.
